# Quinone-Derived π-Extended Phenazines as New Fluorogenic Probes for Live-Cell Imaging of Lipid Droplets

**DOI:** 10.3389/fchem.2018.00339

**Published:** 2018-08-13

**Authors:** Fabio de Moliner, Aaron King, Gleiston G. Dias, Guilherme F. de Lima, Carlos A. de Simone, Eufrânio N. da Silva Júnior, Marc Vendrell

**Affiliations:** ^1^Medical Research Council Centre for Inflammation Research, The University of Edinburgh, Edinburgh, United Kingdom; ^2^Institute of Exact Sciences, Department of Chemistry, Federal University of Minas Gerais, Belo Horizonte, Brazil; ^3^Institute of Physics, University of São Paulo, São Carlos, Brazil

**Keywords:** fluorescence, lapachones, bioimaging, phenazines, lipids

## Abstract

We describe a new synthetic methodology for the preparation of fluorescent π-extended phenazines from the naturally-occurring naphthoquinone lapachol. These novel structures represent the first fluorogenic probes based on the phenazine scaffold for imaging of lipid droplets in live cells. Systematic characterization and analysis of the compounds *in vitro* and in cells led to the identification of key structural features responsible for the fluorescent behavior of quinone-derived π-extended phenazines. Furthermore, live-cell imaging experiments identified one compound (P1) as a marker for intracellular lipid droplets with minimal background and enhanced performance over the lipophilic tracker Nile Red.

## Introduction

**Scheme 1:**
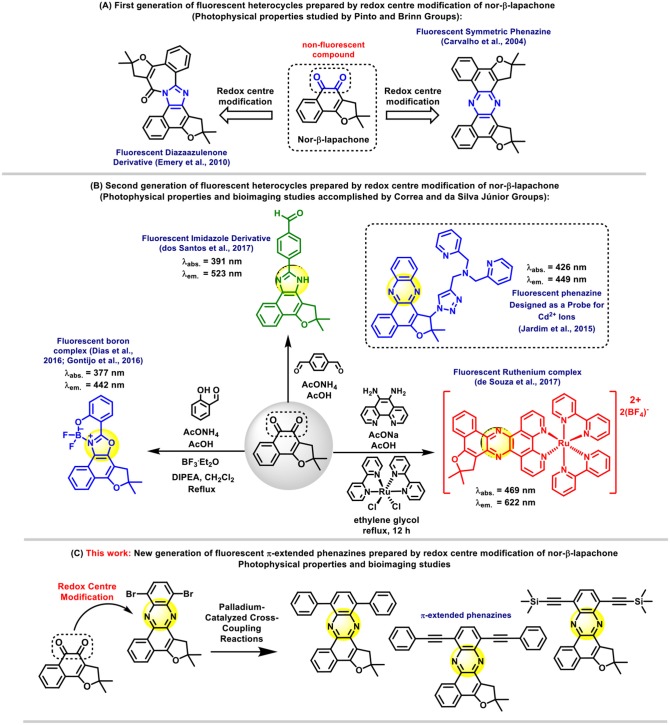
Overview of nor-β-lapachone derivatives and the design of π-extended phenazines for bioimaging applications.

Lipid droplets (LDs) are ubiquitous intracellular organelles which represent the main storage of fatty acids and neutral lipids within the cytoplasm (Martin and Parton, 2006; Farese and Walther, 2009). As such, they display the unique feature of containing highly hydrophobic phases within the aqueous environment of the cytosol (Walther and Farese, 2012). While they were first identified by light microscopy in the 19th century, they were considered structures of little functional importance until the discovery of perilipin (Greenberg et al., 1991), a protein that coats the surface of LDs and is key in the regulation of cell metabolism (Brasaemle et al., 2009). Ever since, interest in their biology and mechanistic dynamics, as well as their interactions with other intracellular organelles and functions in both health and disease has steadily increased over the last decades (Thiele and Spandl, 2008). In particular, LDs have a close relationship with endoplasmic reticulum, in a fashion which strongly points at the existence of an extensive lipids trafficking between these two subcellular compartments (Robenek et al., 2006). This phenomenon involves complex mechanisms that still need to be fully elucidated (Martin et al., 2005). Mitochondria (Blanchette-Mackie and Scow, 1983), peroxisomes (Binns et al., 2006), and endosomes (Liu et al., 2007) have also been proven to associate with LDs to perform several tasks related to lipid metabolism. Alterations in LDs physiology are featured in several pathological conditions, such as obesity (Chen et al., 2002), diabetes (Guilherme et al., 2008), atherosclerosis (Maxfield and Tabas, 2005), fatty liver disease (Fon Tacer and Rozman, 2011), and cancer (Liu et al., 2017). Therefore, the detection of LDs in live-cell imaging has emerged as a powerful tool in biomedical research. Fluorescent probes are valuable and versatile tools for the visualization of intracellular biomolecules and structures, as well as for the differentiation of cell types (Vendrell et al., 2011; Kielland et al., 2012; Er et al., 2013; Park et al., 2014; Hirayama et al., 2016; Zhang et al., 2016; Fernandez et al., 2017; Dias et al., 2018). Unsurprisingly, some hydrophobic dyes such as BODIPY (Karolin et al., 1994) and Nile Red (Greenspan et al., 1985) have been used to stain LDs, but they both display some drawbacks such as limited specificity and short Stokes shifts; hence, the development of novel probes for LDs is an ongoing challenge within the chemical biology community (Cao Y. et al., 2017; Collot et al., 2018). In this context, environmentally-sensitive fluorophores which display enhanced emission in non-polar environments are ideal (Nitz et al., 2002; Vázquez et al., 2005; Shieh et al., 2015; Mendive-Tapia et al., 2016, 2017; Subiros-Funosas et al., 2017; Aron et al., 2018; Kuriki et al., 2018). Since the design of innovative and often unconventional synthetic approaches is often necessary to assemble new fluorescent scaffolds (De Moliner et al., 2017), we envisioned a strategy exploiting the potential of natural products as advanced intermediates toward the generation of phenazine probes for LDs (Scheme 1) (Carvalho et al., 2004; Emery et al., 2010; Dias et al., 2016; Gontijo et al., 2016; de Souza et al., 2017). As a part of our ongoing interest in medicinal chemistry (Yraola et al., 2004; Vendrell et al., 2007, 2009) and the synthesis and application of novel fluorescent probes for imaging of subcellular organelles (Jardim et al., 2015; dos Santos et al., 2017) phenazines **P1** and **P3-P7** were prepared from the naturally-occurring lapachol. Herein we report the chemical synthesis and photophysical characterization of this new set of compounds as well as cell imaging experiments that demonstrate their suitability for the fluorescence staining of LDs in live cells.

## Materials and methods

### Chemistry

#### Reagents and general methods

Starting materials available from commercial suppliers were used as received, unless otherwise stated. Catalytic reactions were performed under an atmosphere of dry nitrogen or argon. Glassware, syringes and needles were either flame dried immediately prior to use or placed in an oven (200°C) for at least 2 h, and allowed to cool either in a desiccator or under an atmosphere of nitrogen or argon. Liquid reagents, solutions or solvents were added via syringe through rubber septa. Melting points were obtained on a Thomas Hoover apparatus and are uncorrected. Column chromatography was performed on silica gel (Silica Flash G60 UltraPure 60–200 μm, 60 Å). Infrared spectra were recorded on an FTIR Spectrometer IR Prestige-21-Shimadzu. ^1^H and ^13^C NMR were recorded at r.t. using a Bruker AVANCE DRX200 and DRX400 MHz instrument in the solvents indicated. Chemical shifts (δ) are given in parts per million (ppm) and coupling constants (J) in Hertz (Hz). Mass spectra were recorded using a Bruker Daltonics FT-ICRMS Apex 4e 7.0T FT-MS (ESI+ mode) and Shimadzu GCMS QP2010+ (EI+ mode). Data were processed employing Bruker Data Analysis software version 4.0. Compounds were named following IUPAC rules as applied by ChemBioDraw Ultra (version 12.0).

#### General procedure for the extraction of lapachol (1) from the heartwood of tabebuia sp. (Tecoma)

In this work, lapachol was extracted from wood that was purchased from local resources in Brazil. Alternatively, lapachol is also commercially available from Sigma-Aldrich. A saturated aqueous sodium carbonate solution was added to the sawdust of the ipê tree. Upon observing the rapid formation of the lapachol sodium salt, hydrochloric acid was added, allowing the precipitation of lapachol. Then, the solution was filtered off and a yellow solid was obtained. This solid was purified by recrystallization with hexane (Ferreira, 1996).

#### Nor-lapachol (2)

The naphthoquinone (**2**) was synthesized by Hooker oxidation methodology and isolated as an orange solid (160 mg, 0.7 mmol, 70% yield). Characterization data are consistent with those reported in the literature (da Rocha et al., 2014; Dias et al., 2015). m.p. 121–122°C. ^1^H NMR (400 MHz, CDCl_3_,): δ = 8.13 (ddd, *J* = 7.5, 1.5, 0.5 Hz, 1 H), 8.10 (ddd, *J* = 7.5, 1.5, 0.5 Hz, 1 H), 7.76 (td, *J* = 7.5, 7.5, 1.5 Hz, 1 H), 7.69 (td, *J* = 7.5, 7.5, 1.5 Hz, 1 H), 6.03–5.99 (m, 1 H), 2.0 (d, *J* = 1.5 Hz, 3 H), 1.68 (d, *J* = 1.2 Hz, 3 H). ^13^C NMR (100 MHz, CDCl_3_,): δ = 184.7, 181.5, 151.1, 143.6, 134.9, 133.0, 132.9, 129.5, 126.9, 126.0, 120.9, 113.6, 26.5, 21.7.

#### Nor-β-lapachone (3)

Sulfuric acid was added dropwise to nor-lapachol (**2**) (4 mmol, 912 mg) until complete dissolution of the quinone. Ice and water were added to solution and the precipitate formed was filtered off and washed with water and then purified by column chromatography on silica gel. The product was eluted in 12% of ethyl acetate in hexane (830 mg, 3.6 mmol, 91% yield, m.p.: 169–171°C). ^1^H NMR (400 MHz, CDCl_3_,) δ: 8.05–803 (m, 1 H), 7.66–7.52 (m, 3 H), 2.93 (s, 2 H), 1.60 (s, 6 H). ^13^C NMR (100 MHz, CDCl_3_,) δ: 181.3, 175.6, 168.7, 134.4, 131.8, 130.9, 129.2, 127.9, 124.5, 115.0, 93.7, 39.3, 28.4.

#### 4,7-dibromo-benzothiadiazole (5)

In a 100 mL three-necked round-bottomed flask, 15 mL of 47% HBr were added to 1.36 g of benzothiadiazole (BTD) (**4**) (10 mmol). The flask was heated to reflux temperature and 10 mL of a 1:1 mixture of Br_2_ and 47% HBr were added dropwise via an addition funnel over 1 h. Further 10 mL of 47% HBr were then added to the reaction mixture all at once and the reflux was maintained for 6 h. Afterwards, it was cooled to r.t. and a saturated solution of NaHSO_3_ was added. The reaction was stirred at r.t. for additional 30 min and then the crude mixture was filtered off and the white solid obtained thereof was washed with acetone (2.35 g, 8 mmol, 80% yield, melting point: 190–191°C). Characterization data are consistent with those reported in the literature (Mancilha et al., 2006). ^1^H NMR (400 MHz, CDCl_3_,) δ: 7.68 (s, 2H). ^13^C NMR (100 MHz, CDCl_3_,) δ: 153.2, 132.6, 114.2.

#### 3,6-dibromobenzene-1,2-diamine (6)

Compound **6** was prepared using the method described by Neto and co-workers with minor modifications (Neto et al., 2005a). In a 50 mL round bottom flask, containing 20 mL of ethanol, 294 mg (1 mmol) of 4,7-dibromo-benzothiadiazole (**5**) were added and heated to reflux. Then, 76 mg (2 mmol) of NaBH_4_ and 2 mg (0.01 mmol) of ${\text{CoCl}}_{2}^{.}$6H_2_O were added. The mixture was refluxed for 2 h, cooled down to r.t. and then filtered off. The solvent was evaporated, water (100 mL) was added, and the organic product was extracted with Et_2_O (3 × 30 mL). The combined organic extracts were dried over Na_2_CO_3_ and the solvent removed, affording the crude product. Due to its instability, the diamine was used in the following reaction without further purification.

#### 2,2-dimethyl-1,2-dihydrobenzo[*a*]furo[2,3-*c*]phenazine (P1)

In a 10 mL flask, nor-β-lapachone (0.7 mmol, 159 mg), *ortho*-phenylenediamine (0.9 mmol, 97 mg), and sodium acetate (0.7 mmol, 57 mg) were added in 3 mL of glacial acetic acid. The reaction was carried out at r.t. upon stirring with a magnetic bar and monitored by thin layer chromatography. After 5 h, all nor-β-lapachone was consumed and the reaction was poured into water with ice. The precipitate was filtered off and then purified by column chromatography on silica gel. The product was eluted with 3% of ethyl acetate in hexane. (200 mg, 0.66 mmol, 95% yield, m.p.: 161–162°C). ^1^H NMR (400 MHz, CDCl_3_): δ = 1.68 (s, 6 H), 3.54 (s, 2 H), 7.67–7.76 (m, 4 H), 8.03 (t, *J* = 4 Hz, 1 H), 8.15 (d, *J* = 8 Hz, 1 H), 8.25 (d, *J* = 8 Hz, 1 H), 9.31–9.33 (m, 1 H). ^13^C NMR (100 MHz, CDCl_3_): δ = 28.4, 41.8, 90.1, 112.8, 122.4, 125.1, 126.0, 127.9, 128.1, 128.5, 129.6, 128.8, 130.0, 132.0, 157.6. IR (cm^−1^): 3,130 (*C* = N) EI/HRMS (m/z): [M+H]^+^: 301.1335. Calcd. for [C_20_H_17_N_2_O]^+^: 301.1341.

#### 9,12-dibromo-2,2-dimethyl-1,2-dihydrobenzo[*a*]furo[2,3-*c*]phenazine (P2)

In a 25 mL flask, nor-β-lapachone (**3**) (1.0 mmol, 228 mg), 3,6-dibromobenzene-1,2-diamine (**6**) (0.9 mmol, 97 mg) and sodium acetate (1.0 mmol, 82 mg) were added in 6 mL of glacial acetic acid. The reaction was carried out at r.t. upon stirring with a magnetic bar and monitored by thin layer chromatography. After 24 h, the reaction was poured into water with ice. The precipitate was filtered off and then purified by column chromatography on silica gel. The product was eluted with 10% of ethyl acetate in hexane. (223 mg, 0.49 mmol, 49% yield, melting point: 227–228°C). ^1^H NMR (400 MHz, CDCl_3_): δ = 1.69 (s, 6 H), 3.58 (s, 2 H), 7.77–7.82 (m, 2 H), 7.85 (d, *J* = 8 Hz, 1 H), 7.92 (d, *J* = 8 Hz, 1 H), 8.06 (t, *J* = 4 Hz, 1 H), 9.40 (t, *J* = 4 Hz, 1 H). ^13^C NMR (100 MHz, CDCl_3_): δ = 29.0, 41.6, 91.2, 113.0, 122.7, 123.1, 124.8, 125.5, 127.0, 128.8, 130.6, 130.9, 131.5, 132.9, 137.6, 140.8, 142.0, 144.0, 159.0. IR (cm^−1^): 1030 (C-Br). EI/HRMS (m/z): [M+H]^+^: 456.9531. Calcd. for [C_20_H_15_Br_2_N_2_O]^+^: 456.9551.

#### 2,2-dimethyl-9,12-diphenyl-1,2-dihydrobenzo[*a*]furo[2,3-*c*]phenazine (P3)

In a Schlenk tube, phenazine dibromide (**P2**) (0.25 mmol, 115 mg), phenylboronic acid (1.0 mmol, 122 mg), Pd(OAc)_2_ (0.05 mmol, 12 mg), triphenylphosphine (0.03 mmol, 10 mg), 2 mL of 2M Na_2_CO_3_ solution were added. The tube was sealed and the reaction was carried out at 70°C upon stirring with a magnetic bar by 36 h. After that, the reaction was cooled to r.t., filtered through Celite and then purified by column chromatography on silica gel. The product was eluted with 5% of ethyl acetate in hexane (91 mg, 0.20 mmol, 80% yield, m.p.: 247–248°C). ^1^H NMR (400 MHz, CDCl_3_): δ = 1.57 (s, 6 H), 3.55 (s, 2 H), 7.35–7.47 (m, 4 H), 7.51 (t, *J* = 8 Hz, 2 H), 7.59–7.66 (m, 2 H), 7.78 (d, *J* = 8 Hz, 1 H), 7.84 (d, *J* = 8 Hz, 1 H), 7.86–7.89 (m, 4 H), 7.95 (d, *J* = 2 Hz, 1 H). ^13^C NMR (100 MHz, CDCl_3_): δ = 28.9, 41.5, 90.2, 113.4, 122.3, 125.3, 126.4, 127.4, 127.5, 127.9, 128.0, 128.1, 129.5, 129.9, 131.3, 131.4, 132.3, 137.9, 139.0, 139.1, 139.3, 140.1, 140.4, 140.9, 142.3, 155.5, and 157.6. IR (cm^−1^): 3135 (*C* = *N*), 1600, 1500. EI/HRMS (m/z) [M+H]^+^: 453.1961. Calcd. for [C_32_H_25_N_2_O]^+^: 453.1967.

#### General procedure for sonogashira reactions

In a Schlenk tube, phenazine dibromide (**P2**) (0.25 mmol, 115 mg), Pd(PPh_3_)_2_Cl_2_ (0.06 mmol, 45 mg) and CuI (0.06 mmol, 11 mg) were added. The tube was vented and filled with nitrogen three times. Alkyne (1.0 mmol), 3 mL of dried toluene and 3 mL of dried triethylamine were added via a syringe. The tube was sealed and the reaction was carried out at 100°C upon stirring with a magnetic bar for 48 h. After that, reactions were cooled to r.t., filtered through Celite and crudes were purified by column chromatography on silica gel with ethyl acetate and hexane.

#### 2,2-dimethyl-9,12-bis[(trimethylsilyl)ethynyl]-1,2-dihydrobenzo[*a*]furo[2,3-*c*]phenazine (P4)

Yellow solid, 103 mg, 0.21 mmol, 82% yield, m.p.: 156–159°C. ^1^H NMR (400 MHz, CDCl_3_): δ = 0.37 (s, 9 H), 0.42 (s, 9 H), 1.69 (s, 6 H), 3.56 (s, 2 H), 7.76–7.81 (m, 2 H), 9.40–9.44 (m, 1 H), 9.42 (dd, *J* = 3.6 and 3.2 Hz, 1 H). ^13^C NMR (100 MHz, CDCl_3_): δ = 0.1, 0.2, 28.7, 90.6, 102.0, 102.1, 103.0, 103.2, 113.1, 122.4, 122.7, 124.1, 125.3, 126.0, 126.4, 127.1, 128.2, 129.8, 130.0, 130.8, 131.4, 131.8, 133.4, 134.2, 139.7, 141.3, 142.7, 143.5, 158.6. IR (cm^−1^): 3252 (CH_3_) EI/HRMS (m/z): [M]^+^: 492.2055. Calcd for [C_30_H_32_N_2_OSi_2_]^+^: 492.2053.

#### 9,12-diethynyl-2,2-dimethyl-1,2-dihydrobenzo[*a*]furo[2,3-*c*]phenazine (P5)

In a 10 mL flask, TMS-phenazine **P4** (0.15 mmol, 75 mg) and potassium fluoride (0.60 mmol, 45 mg) were added in methanol (5 mL). The reaction was carried by magnetic stirring at r.t. for 48 h. Compound **P5** was purified by column chromatography on silica gel. The product was eluted with 3% of ethyl acetate in hexane. (yellow solid, 70 mg, 0.14 mmol, 95% yield, m.p.: 195–197°C). ^1^H NMR (400 MHz, CDCl3): δ = 1.68 (s, 6 H,), 3.59 (s, 2 H), 3.64 (s, 1 H), 3.72 (s, 1 H), 7.77–7.80 (m, 2 H), 7.87 (d, *J* = 8 Hz, 1 H), 7.94 (d, *J* = 8 Hz, 1 H), 8.06–8.08 (m, 1 H), 9.41–9.44 (m, 1 H). ^13^C NMR (100 MHz, CDCl_3_): δ = 29.9, 41.4, 80.9, 84.8, 85.0, 90.8, 113.2, 122.4, 122.6, 123.8, 125.4, 126.7, 128.5, 130.3, 131.8, 131.9, 134.0, 139.7, 141.7, 142.8, 143.8 and 158.9. IR (cm^−1^): 3135 (*C* = *N*); 2953, 2149, 852. EI/HRMS (m/z) [M+H]^+^: 349.1335. Calcd for [C_24_H_17_N_2_O]^+^: 349.1341.

#### 2,2-dimethyl-9,12-bis(phenylethynyl)-1,2-dihydrobenzo[*a*]furo[2,3-*c*]phenazine (P6)

Compound **P6** was prepared following the procedure described above. Yellow solid, 75 mg, 0.15 mmol, 69% yield, m.p.: 212–215°C. ^1^H NMR (400 MHz, CDCl_3_): δ = 1.70 (s, 6 H), 3.62 (s, 2 H), 7.38–7.44 (m, 6 H), 7.70 (d, *J* = 1.6 Hz, 1 H), 7.72 (d, *J* = 1.6 Hz, 1 H), 7.76–7.80 (m, 4 H), 7.89 (d, *J* = 7.6 Hz, 1 H), 7.96 (d, *J* = 7.6 Hz, 1 H), 8.05–8.07 (m, 1 H), 9.44 (dd, *J* = 5.6 and *J* = 1.2 Hz, 1 H). ^13^C NMR (100 MHz, CDCl_3_): δ = 29.0, 41.4, 87.5, 87.6, 90.8, 97.5, 97.7, 113.4, 122.6, 122.9, 123.9, 124.3, 125.4, 126.5, 128.3, 128.5, 128.6, 128.7, 128.8, 130.2, 131.3, 132.0, 132.2, 133.2, 139.7, 141.4, 142.8, 143.6, 158.7. IR (cm^−1^): 3135 (*C* = N). EI/HRMS (m/z): [M+H]^+^: 501.1961. Calcd for [C_36_H_25_N_2_O]^+^: 501.1967.

#### 9,12-bis[(4-methoxyphenyl)ethynyl]-2,2-dimethyl-1,2-dihydrobenzo[*a*]furo[2,3-*c*]phenazine (P7)

Compound **P7** was prepared following the procedure described above. Yellow solid, 66 mg, 0.17 mmol, 71% yield, m.p.: 254–255°C. ^1^H NMR (400 MHz, CDCl_3_): δ = 1.70 (s, 6H); 3.62 (s, 2 H); 3.84 (s, 3 H), 3.86 (s, 3 H), 6.63 (d, *J* = 8 Hz, 2 H), 6.97 (d, *J* = 12 Hz, 2 H). 7.64 (d, *J* = 8 Hz, 2 H), 7.71 (d, *J* = 8 Hz, 2 H), 7.76–7.82 (m, 2 H); 7.87 (d, *J* = 8 Hz, 1 H), 7.94 (d, *J* = 8 Hz, 1 H), 8.07 (d, *J* = 7 Hz, 1 H), 9.47 (d, *J* = 8 Hz, 1 H). ^13^C NMR (100 MHz, CDCl_3_): δ = 28.9, 41.4, 55.5, 86.3, 86.4, 90.6, 97.5, 97.7, 113.3, 114.2, 114.3, 116.0, 122.5, 122.8, 124.2, 125.4, 126.4,128.3, 130.0, 130.9, 132.0, 132.8, 133.6, 139.6, 141.2, 142.7, 143.4, 158.5, 160.0, 160.1 and 160.2. IR (cm^−1^): 3,135 (*C* = N); 1248, 1040. EI/HRMS (m/z) [M+H]^+^: 561.2166. Calcd for [C_38_H_29_N_2_O_3_]^+^: 561.2179.

### Spectral characterization

Spectroscopic and quantum yield data were recorded on a Synergy HT spectrophotometer (Biotek). Compounds were dissolved at the indicated concentrations and spectra were recorded at r.t. Spectra are represented as means from at least two independent experiments with *n* = 3. Quantum yields were calculated by measuring the integrated emission area of the fluorescence spectra and comparing it to the area measured for fluorescein in basic EtOH.

### Cell imaging

HeLa cells were grown in DMEM cell culture media supplemented with 10% FBS, antibiotics (100 U mL^−1^ penicillin, 100 mg mL^−1^ streptomycin) and 2 mM L-glutamine in a humidified atmosphere at 37°C with 5% CO_2_. Cells were plated on glass chamber slides Lab-Tek™ II (Nunc) the day before the imaging experiment. Cells were incubated with compounds at the indicated concentrations at 37°C for 15 min, washed with PBS, and imaged under a EVOS FL2 epifluorescence microscope equipped with a live cell imaging stage. Fluorescence images were acquired using a 40X oil objective. All images were analyzed and processed with ImageJ.

### X-ray analysis

X-ray diffraction data collection for the compound was performed on an Enraf-Nonius Kappa-CCD diffractometer (95 mm CCD camera on κ-goniostat) using graphite monochromated MoK_radiation (0.71073 Å) at r.t. Data collection was carried out using the COLLECT software (Enraf-Nonius COLLECT; Nonius BV: Delft, The Netherlands, 1997–2000) up to 50° in 2θ. Integration and scaling of the reflections, correction for Lorentz and polarization effects were performed with the HKL DENZO-SCALEPACK system of programs (Otwinowski and Minor, 1997). The structure of the compound was solved by direct methods with SHELXS-97 (Sheldrick, 1997a). The model was refined by full-matrix least squares on F^2^ using the SHELXL-97 (Sheldrick, 1997b). The program ORTEP-3 (Farrugia, 1997) was used for graphic representation and the program WINGX (Farrugia, 1999) for presentation purposes. All H atoms were located by geometric considerations (C–H = 0.93–0.97 Å) and refined as riding with Uiso(H) = 1.5Ueq(C-methyl) or 1.2Ueq(other). The reference number for the compound is CCDC 1841868. Copies of the available material can be obtained, free of charge on application to the Director, CCDC, 12 Union Road, Cambridge CH21EZ, UK (fax: +44-1223-336-033 or e-mail: deposit@ccdc.cam.ac.uk or http://www.ccdc.cam.ac.uk).

### Particle size analysis

The mean aggregate diameters in aqueous media were determined by dynamic light scattering using a PS90 Particle Size Analyser (Brookehaven Instrument Corporation). Compounds **P1** and **P3–P7** were dissolved in PBS (10 μM) and kept at r.t. to measure the aggregate size after 10 min. Data is represented as means ± s.e.m from two independent experiments (*n* = 5).

## Results and discussion

### Chemical synthesis

**Scheme 2:**
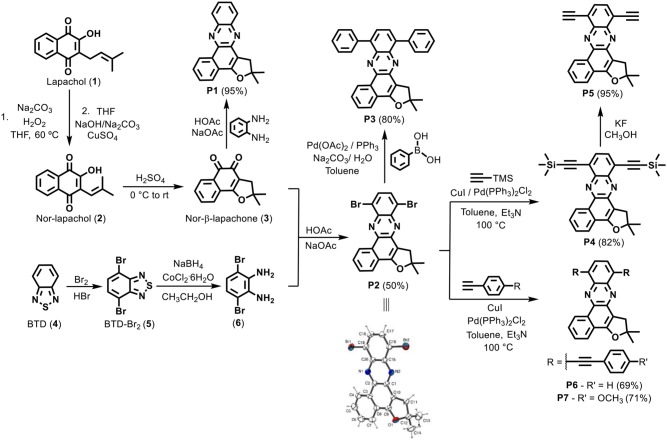
Synthetic scheme for phenazine derivatives **P1**–**P7** and crystal structure of compound **P2**.

The new fluorescent π-extended phenazines were prepared according to the synthetic procedures shown in Scheme 2. Our strategy was based on the preparation of dibromophenazine **P2**, which was further subjected to Pd-catalyzed cross-coupling reactions aiming to produce π-extended derivatives **P3**–**P7**. Compound **P1** was instead obtained through a straightforward condensation between nor-β-lapachone and *ortho*-phenylenediamine. All these fluorescent probes were obtained from lapachol (**1**), a naturally-occurring naphthoquinone that can be extracted from the heartwood of *Tabebuia sp*. (Tecoma) (Ferreira, 1996). This plant material, which is known as ipê wood, is widely available throughout South America, where it can be easily sourced from a variety of trees and purchased from commercial sources. With compound **1** in hand, nor-lapachol (**2**) was prepared via Hooker oxidation (Fieser and Fieser, 1948; Lee et al., 1995; Eyong et al., 2012). The treatment of compound **2** with sulphuric acid then afforded nor-β-lapachone **3**. In parallel, 3,6-dibromobenzene-1,2-diamine (**6**) was prepared via a well-established synthetic route described by Neto et al. (2005a). Initially, 4,7-dibromo-2,1,3-benzothiadiazole (**5**) was prepared from 2,1,3-benzothiadiazole (**4**) following a previously reported methodology with minor modifications (Neto et al., 2013). The sulfur extrusion from compound **5** was accomplished with NaBH_4_ in the presence of catalytic amounts of CoCl_2_•6H_2_O (1 mol%) (Neto et al., 2005b). Subsequently, the reaction between compound **6** and nor-β-lapachone **3** provided the key intermediate phenazine **P2** in moderate yields.

Compound **P2** was used in the preparation of all the phenazine probes herein described, except for **P1**. Its structure was unambiguously determined by crystallographic analysis upon recrystallization, with high-quality crystals being obtained by slow evaporation of **P2** upon solubilization in dichloromethane. Single crystal X-ray analysis revealed the structure of the compound as displayed in Scheme 2, where the atoms labeling and the displacement ellipsoids at the 50% probability level are shown. Compound **P2** crystallized in the monoclinic P-1 space group with two molecules in the unit cell. Additional details about the data collection and structure refinement are described in the Supporting Information (Table S1). The molecular structure of compound **P2** shows planarity for all six members rings, where the largest deviation [0.058(2) Å] from the least-square plane being exhibited by atom C18. The furan ring has an envelope conformation with the flap (C12) (0.27 Å) above of the plane of the rings and the puckering parameters calculated for this conformation were: *q*_2_ = 0.135(3)Å, and ϕ = 101.9(2)° (Cremer and Pople, 1975).

Next, compound **P3** was prepared in good yields by cross-coupling Suzuki reaction from the intermediate **P2**. Pd-catalyzed reactions were also employed to derivatize the key intermediate to obtain compounds **P4**, **P6**, and **P7**. Sonogashira coupling proved effective for the insertion of terminal alkynes substituted with aryl groups, leading to compounds **P6** and **P7** in 69 and 71% yields, respectively. Finally, compound **P5** was synthesized in almost quantitative yields by removal of the trimethylsilane protecting group with potassium fluoride in methanol.

### Photophysical characterization

Since phenazines **P1** and **P3**–**P7** were designed as potential LD-staining dyes, partly due to their hydrophobicity, screening of their optical properties in different solvents (e.g., aqueous media and organic solvents) was undertaken as a preliminary assessment of their fluorescence behavior. Dioxane was chosen as the organic solvent given its dielectric constant (ε: 2.25), which closely mimics the polarity of lipid-rich cellular compartments, such as phospholipid bilayers with a dielectric constant around 2.0 (Pérochon et al., 1992). The optical readout of phenazines in aqueous media was weaker than in organic solvents. Absorbance maxima were observed in the blue region of the visible spectrum (420–460 nm), regardless of their substitution pattern. Likewise, fluorescence emission spectra for compounds **P1** and **P3**–**P7** were also recorded, with wavelengths and quantum yields being summarized in Table 1.

**Table 1:** Summary of photophysical properties for compounds **P1** and **P3**–**P7**.

**Compound**	**λ_abs._ (nm)^‡^**	**λ_em._ (nm)^‡^**	**Stokes shift (nm)^‡^**	**Q.Y.^‡^**	λ_em._ (nm)^*^	Q.Y.^*^	**Mean aggregate diameter (nm)**
P1	428	500	72	0.22	525	0.04	380 ± 50
P3	437	501	64	0.11	520	0.01	>10,000
P4	451	524	73	0.19	550	0.01	80 ± 10
P5	450	528	78	0.26	560	0.02	640 ± 160
P6	422	527	105	0.21	555	0.01	>10,000
P7	434	530	96	0.25	551	<0.01	>10,000

*Wavelengths determined in dioxane. Quantum yields relative to fluorescein in basic ethanol (Φ = 0.93) (Sjöback et al., 1995)*.

‡ *Determined in dioxane*;

* *Determined in water*.

**Figure 1:**
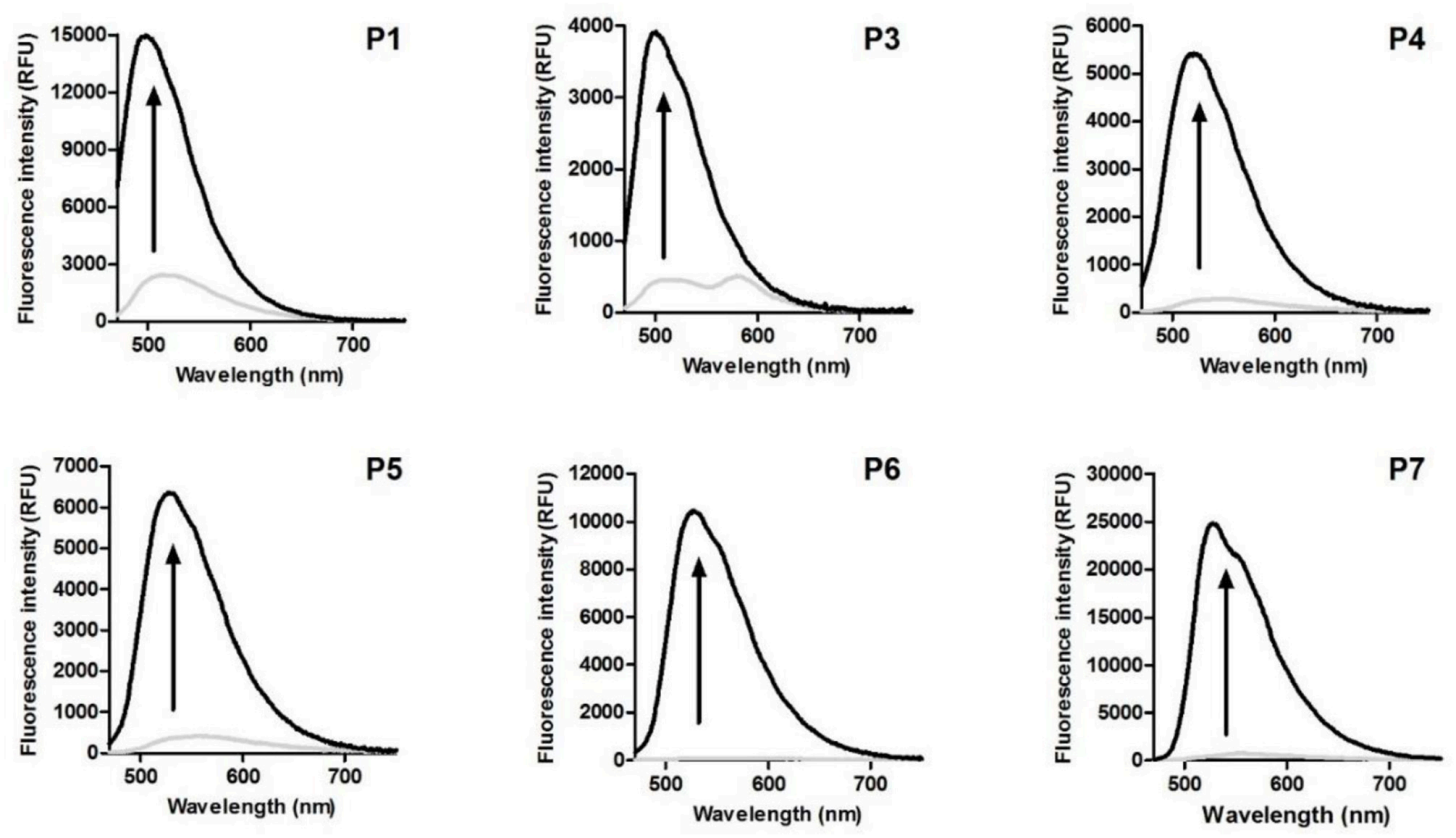
Fluorescence emission spectra of phenazine-derived fluorophores **P1** and **P3**–**P7** in water (gray line) and dioxane (black line). Spectra were recorded at 50 μM. λ_exc.:_ 440 nm.

As shown in Table 1, compounds **P1** and **P3**, which both lack alkyne substituents, displayed shorter emission wavelengths (i.e., 525 and 520 nm, respectively) compared to phenazines with alkyne moieties, which showed red-shifted emission wavelengths around 550–560 nm (Table 1). Fluorescence analysis in dioxane as an organic solvent with decreased polarity exhibited brighter quantum yields for most phenazines as well as blue-shifted emission (e.g., from 500 to 530 nm) maxima for all compounds (Figure 1).

Based on existing literature on environmentally-sensitive fluorophores (Levit et al., 2009; Cao X. et al., 2017), we also decided to examine response to viscosity as it can potentially affect optical properties of the phenazines. Compounds were dissolved in water-glycerol mixtures of increasing viscosity, and their fluorescence intensity was measured. All phenazines showed brighter fluorescence emission in more viscous media, with fold increases ranging from 2 (compound **P3** and **P4**) to 10 (compound **P5**) (Figure S1). Interestingly, compounds **P1** and **P5**, which lack bulky groups attached to the phenazine core, were found to be the most responsive to changes in viscosity.

### Structure-activity relationships

**Figure 2:**
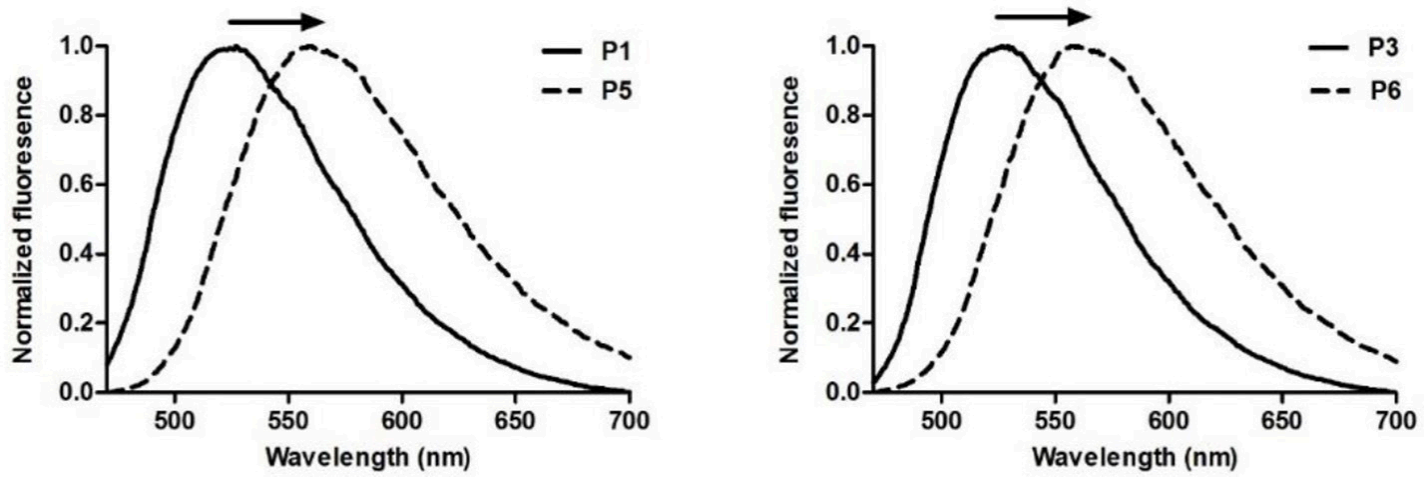
Comparison of normalized emission spectra of phenazine probes without (**P1**, **P3**) and with (**P5**, **P6**) alkyne substitution. Spectra were recorded at 50 μM. λ_exc.:_ 440 nm.

In order to examine the key features of phenazine-based probes **P1** and **P3**–**P7** as fluorophores as well as their capabilities to stain intracellular hydrophobic environments, we evaluated the impact of different structural modifications (e.g., different electron-rich conjugated moieties) on their fluorescence properties. First, we analyzed the influence of C-C triple bonds by comparing the behavior of compounds **P1** vs. **P5** as well as compounds **P3** vs. **P6**. In both cases, the addition of alkyne groups resulted in 30 nm red-shifts of emission maxima [i.e., from 520 to 550 nm (**P3** vs. **P6**) and 525 to 560 nm (**P1** vs. **P5**)] (Figure 2). Since the wavelength of emitted photons is related to the energy gap between the ground and excited electronic state, it can be inferred that triple bonds lower the energy of the excited state of the probes, partially due to a greater delocalization of the electrons across the fluorophore.

Increased sensitivity to polarity was also observed upon addition of substituents bearing triple bonds to the core of the probes. Both compounds **P5** and **P6** proved considerably more sensitive to changes in polarity compared to their analogs lacking alkyne groups. Specifically, compounds **P5** and **P6** showed 9-fold and 21-fold increases respectively in fluorescence quantum yields when comparing water to dioxane. On the other hand, **P1** and **P3** displayed 6-fold and 11-fold increases, respectively.

Next, we also examined the impact of direct C-C coupling of electron-rich phenyl groups to the phenazine core. For this purpose, we compared **P1** vs. **P3** as well as **P5** vs. **P6**. Notably, unlike alkyne substitution, the C-C addition of phenyl groups had minor impact on absorption and emission profiles of the fluorophores. On the other hand, their influence in sensitivity to viscosity was quite pronounced. Whereas, compound **P1** showed 6-fold increase when dissolved in glycerol compared to water, compound **P3** showed only 2-fold increase. A similar trend was observed for compounds **P5** and **P6**, which exhibited 10-fold and 3-fold increases, respectively. Further insight into the impact of electron-donating moieties was gained by evaluating the effect of a methoxy group in the para position of the aromatic ring in compound **P7**. A minor 13 nm red-shift in the absorbance maximum was observed compared to **P6**, with the emission spectra of both probes being identical. Likewise, the response to variations in viscosity was also unaffected by the addition of the methoxy group.

Finally, the optical properties of trimethylsilane-containing compound **P4** were also analyzed. Although trimethylsilyl tags primarily serve as protecting groups for terminal alkynes (Greene and Wuts, 1999), they can also offer insight into the impact of bulky, non-planar substituents on fluorescence. The properties of compound **P4** were compared to its unprotected analog **P5**, showing that trimethylsilyl groups slightly increased environmental sensitivity (i.e., 19-fold increase from water to dioxane for compound **P4**, while **P5** shows a 13-fold increase under the same conditions).

Solubility in aqueous media is an important feature to be considered in the design of fluorescent probes for live-cell imaging. Therefore, we assessed the solubility behavior of compounds **P1** and **P3–P7** in PBS by measuring the mean size of aggregates at room temperature using dynamic light scattering. All compounds endowed with aryl substituents, either directly connected to the phenazine core as in **P3** or linked to it through a triple bond (**P6** and **P7**) produced very large aggregates in aqueous medium, showing potential incompatibility for live-cell imaging. On the other hand, remarkably better water solubility was observed for probes **P1**, **P4**, and **P5**, which display smaller, non-aromatic residues. These results suggest that, even though compounds **P6** and **P7** displayed the highest environment-sensitivity, their poor water solubility may hamper their use for staining intracellular LDs.

### Live-cell imaging

In order to assess the properties of compounds **P1** and **P3**–**P7** for live-cell imaging, we performed fluorescence microscopy experiments in human HeLa cells. Initially, all compounds were individually incubated at 10 μM concentration for 15 min together with the nuclear fluorescent counterstain Hoechst 33342, then washed and imaged under the microscope.

**Figure 3:**
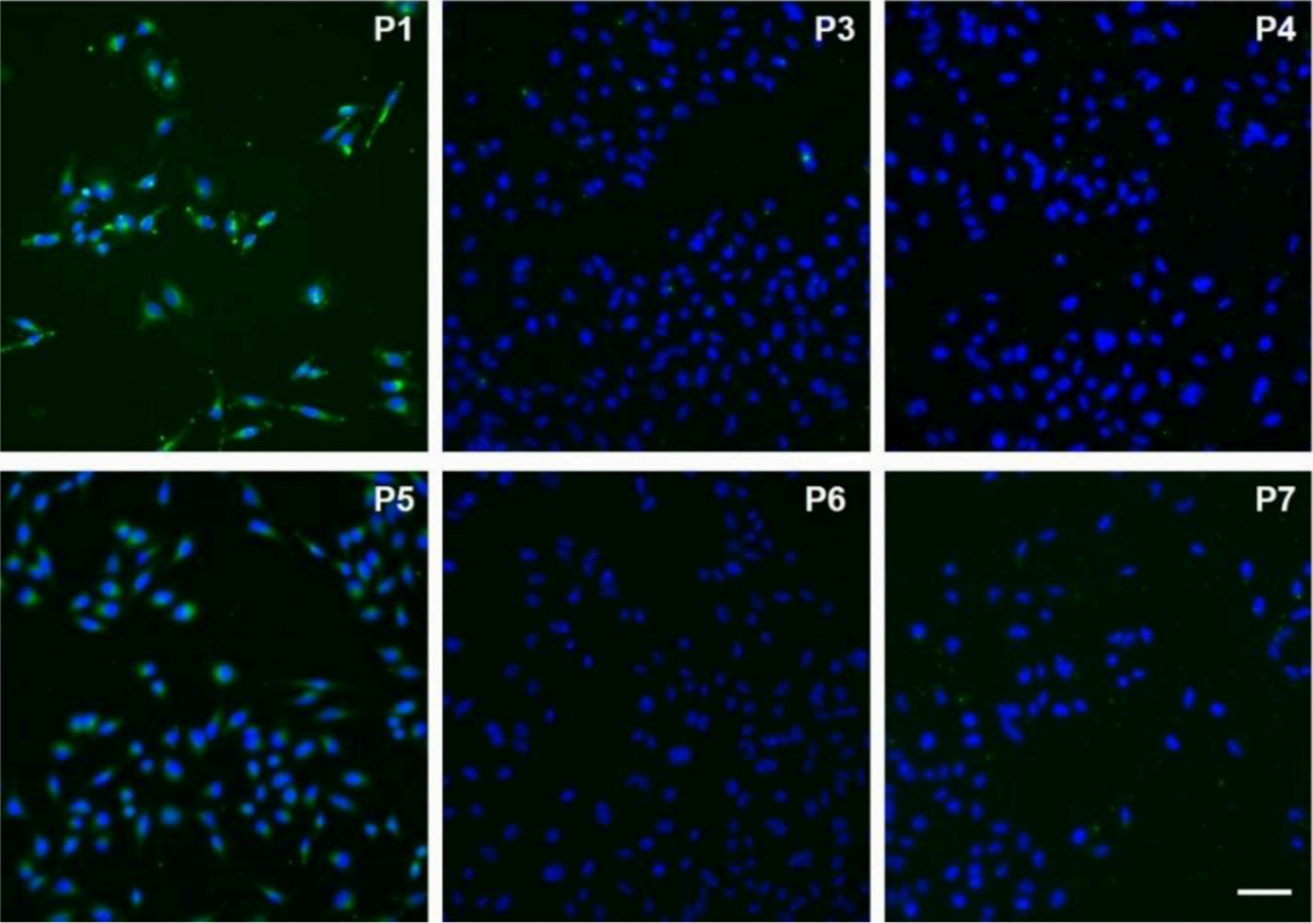
Fluorescence microscopy images of HeLa cells incubated with compounds **P1** and **P3**–**P7** (10 μM, green) for 15 min and counter-stained with Hoechst 33342 (blue). Scale bar: 50 μm.

As shown in Figure 3, fluorescence emission was only detected from cells that had been incubated with compounds **P1** or **P5**, with the former being significantly brighter than the latter. The lack of fluorescence in cells incubated with other phenazines (**P3**, **P4**, **P6**, and **P7**) indicates poor intracellular retention, which -with the exception of compound **P4**- may be due to their scarce water solubility. We further explored the imaging capabilities of **P1** as the most suitable phenazine for fluorescence imaging. We incubated HeLa cells with different concentrations of the phenazine, and observed a dose-dependent response with an optimal working concentration around 10 μM (Figure S2). High-magnification images displayed a distinct punctate pattern in the cytoplasm of **P1**-stained cells, suggesting preferential accumulation at intracellular LDs.

**Figure 4:**
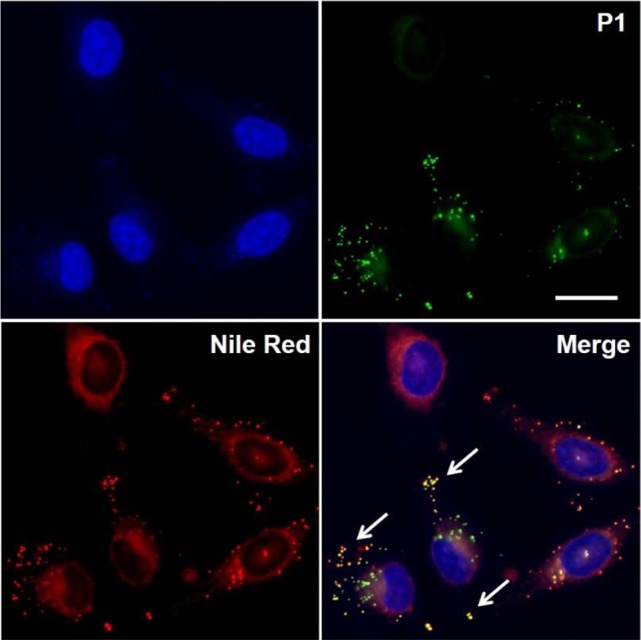
High magnification fluorescence microscopy images of HeLa cells incubated with compound **P1** (10 μM, green), Nile red (10 μM, red) and Hoechst 33342 (blue). Overlaid images indicate co-localization of **P1** and Nile Red in intracellular LDs as shown by punctate yellow signals (white arrows). Scale bar: 10 μm.

In order to further analyze the subcellular localization of compound **P1** and its potential application of LD imaging in live cells, we performed co-localization experiments with the commercially available lipophilic dye Nile Red (λ_em_ > 600 nm), which has been reported for visualizing LDs in cells (Figure S4) (Greenspan et al., 1985). As shown in Figure 4, HeLa cells were incubated with compound **P1** (green), Nile Red (red) and Hoechst 33342 (blue) as the nuclear counterstain. Notably, both compound **P1** and Nile Red were found in the cytoplasm with preferential accumulation in punctate intracellular lipid environments. Overlaying of both channels confirmed the co-localization of **P1** and Nile Red in LDs (yellow dots in Figure 4, white arrows). Furthermore, compound **P1** showed reduced off-target intracellular fluorescence in the cytosol asserting its value as a fluorogenic probe for hydrophobic intracellular environments. Notably, the remarkable environment-sensitivity of compound **P1** allowed us to observe staining of LDs even under wash-free conditions, opening new opportunities for imaging LD dynamics in real time (Figure S3). Altogether, these results validate the utility of compound **P1** as a new cell-permeable fluorophore for live-cell imaging of intracellular LDs.

## Conclusions

In summary, we have prepared a collection of fluorescent phenazine compounds using a novel and straightforward synthetic strategy that exploits chemical modifications on the naturally-occurring lapachol. We characterized their optical and intracellular staining properties, and identified the first fluorogenic phenazine for lipid droplet staining in live cells. Furthermore, we established structure-activity relationships to analyze the influence of phenazine substitution in their fluorescent behavior, and to determine key structural features associated to their fluorescence sensitivity. Furthermore, we assessed the formation of aggregates in aqueous media in order to determine the relationship between the phenazine substitution pattern and their water solubility as one of the key features for applications in live-cell imaging. This systematic study will improve current limitations in the rational design of phenazine fluorogenic agents for bioimaging applications. Finally, we validated the phenazine derivative **P1** as a live-cell compatible fluorescent probe for intracellular staining of lipid droplets with minimal off-target signal and enhanced capabilities over the commercially available dye Nile Red.

## Author contributions

EdSJ and MV conceived the molecules, strategies, and designed the experiments. FdM, AK, GdL, and GD performed the experiments. CdS performed all crystallographic studies. FdM, EdSJ, and MV analyzed the data and wrote the paper.

### Conflict of interest statement

The authors declare that the research was conducted in the absence of any commercial or financial relationships that could be construed as a potential conflict of interest.
