# Supplementary material for: Quinone-Derived π-Extended Phenazines as New Fluorogenic Probes for Live-Cell Imaging of Lipid Droplets
Source: Front Chem. 2018 Aug 13;6:339. doi: 10.3389/fchem.2018.00339 (PMC6099520; doi:10.3389/fchem.2018.00339)
Supplement: Supplementary file 1 [file Data_Sheet_1.PDF]

Electronic Supplementary Information

# Quinone-derived π-extended phenazines as new fluorogenic probes for live-cell imaging of lipid droplets

Fabio de Moliner, Aaron King, Gleiston G. Dias, Guilherme F. de Lima, Carlos A. de Simone, Eufrânio N. da Silva Júnior and Marc Vendrell

**NMR Spectra**

**Lapachol (1)**

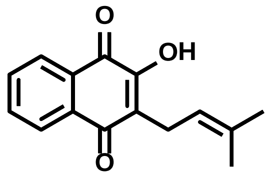


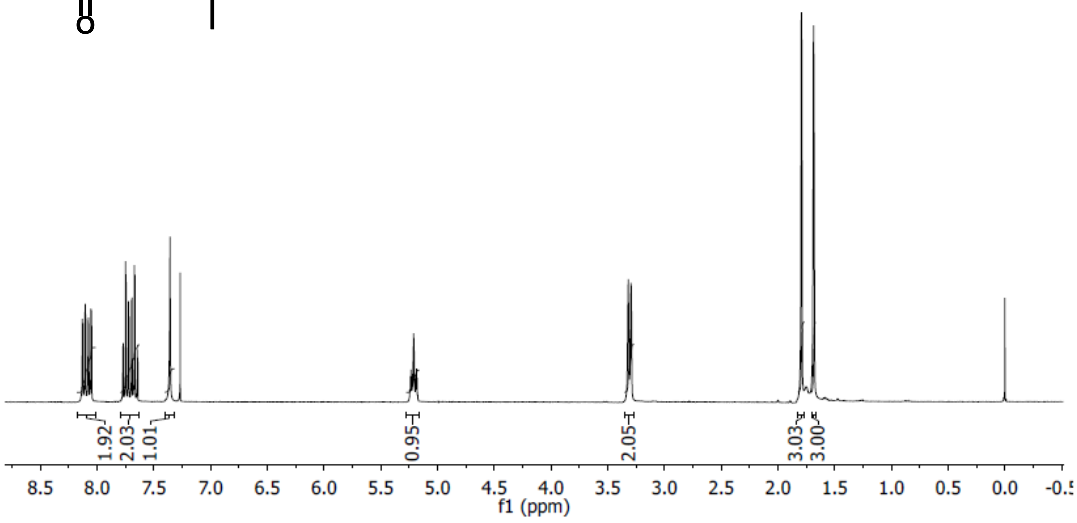


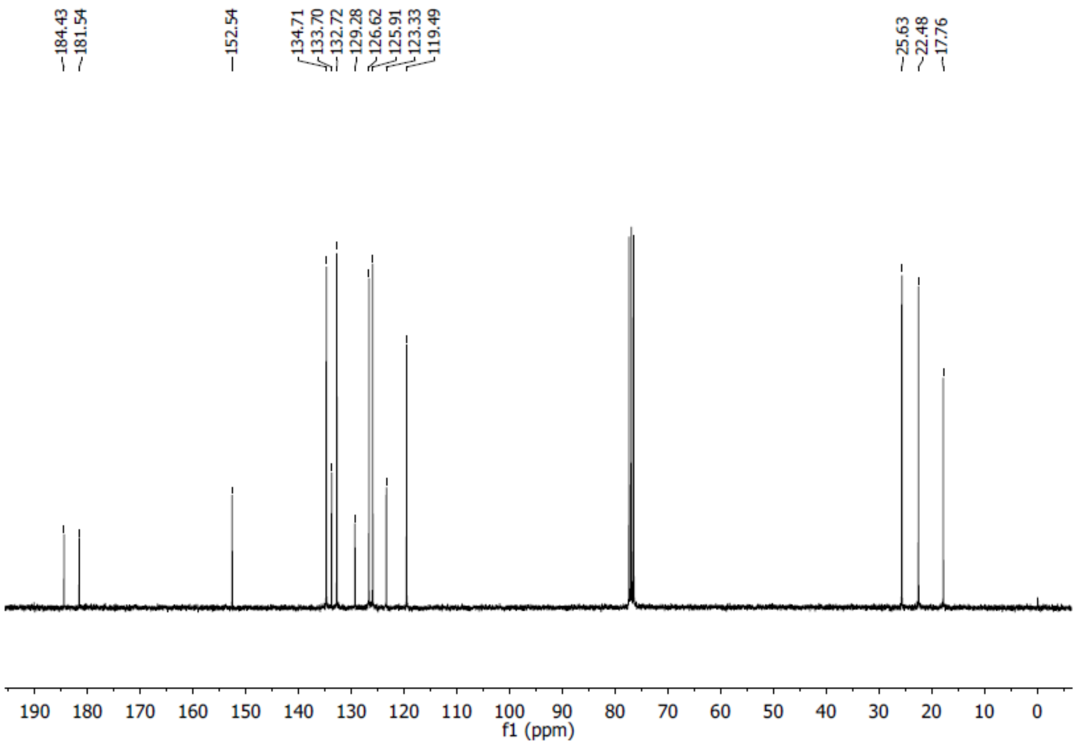

**Nor-lapachol (2)**

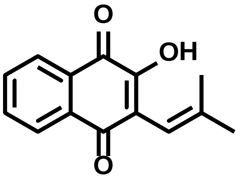


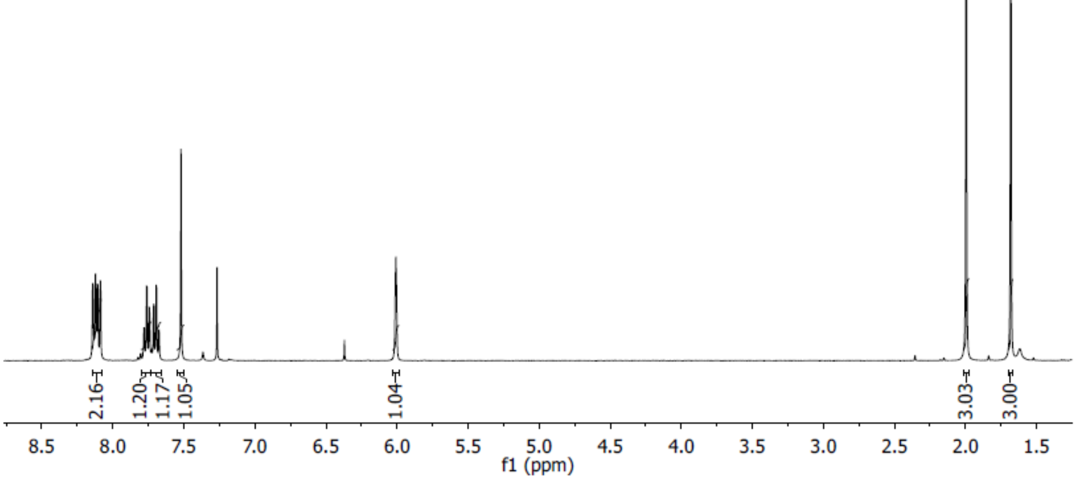


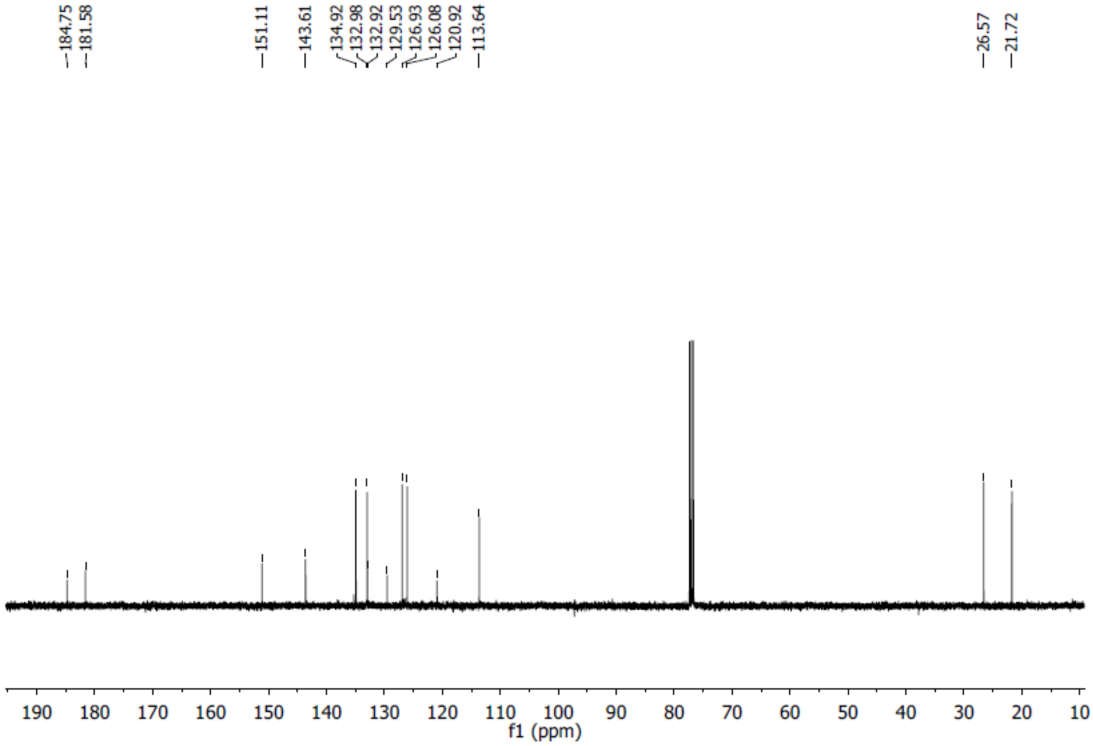

**Nor-β-lapachone (3)**

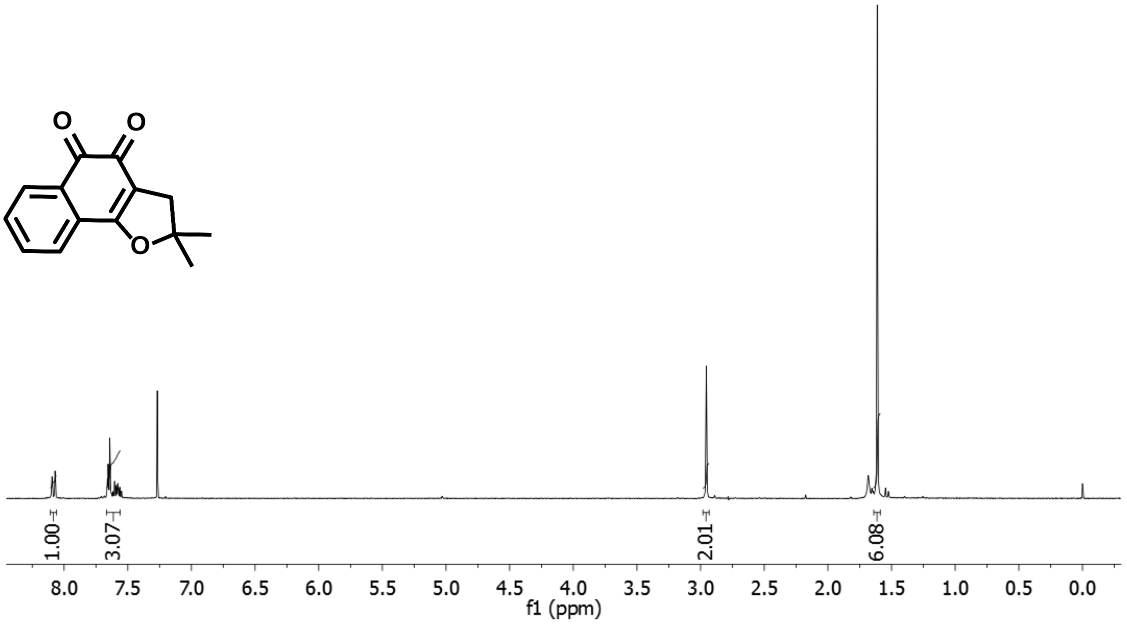


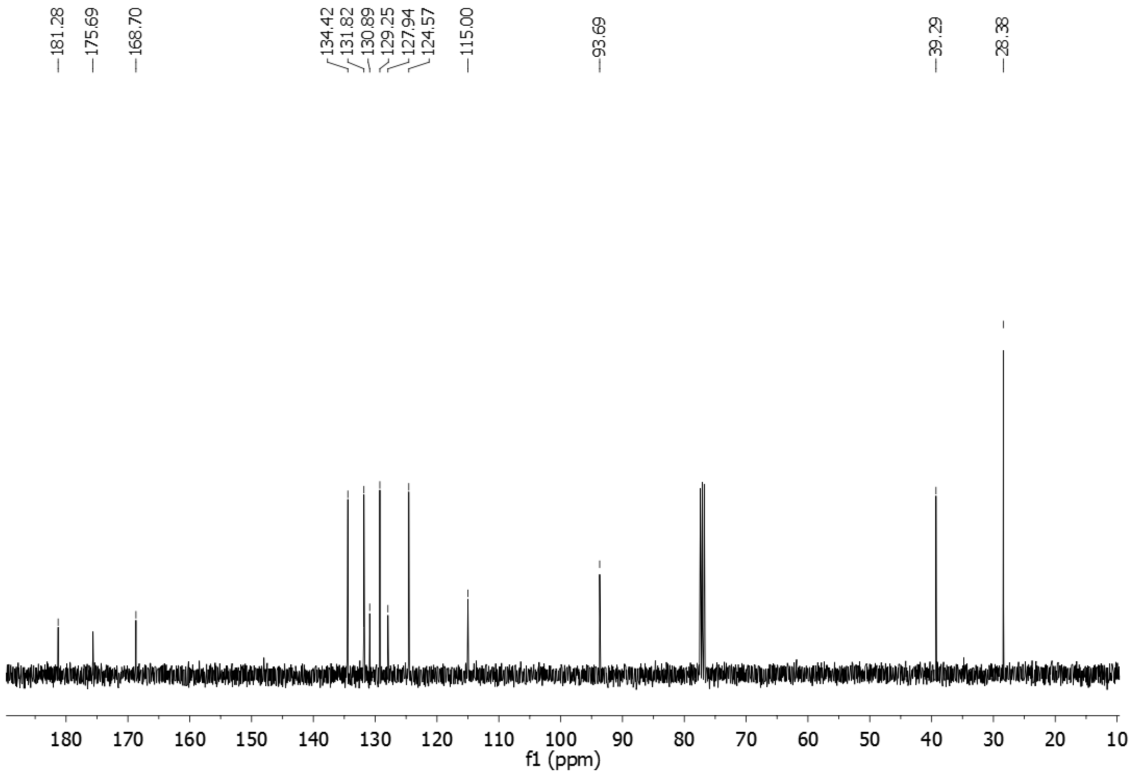

**4,7-Dibromo-benzothiadiazole (5)**

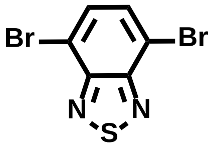


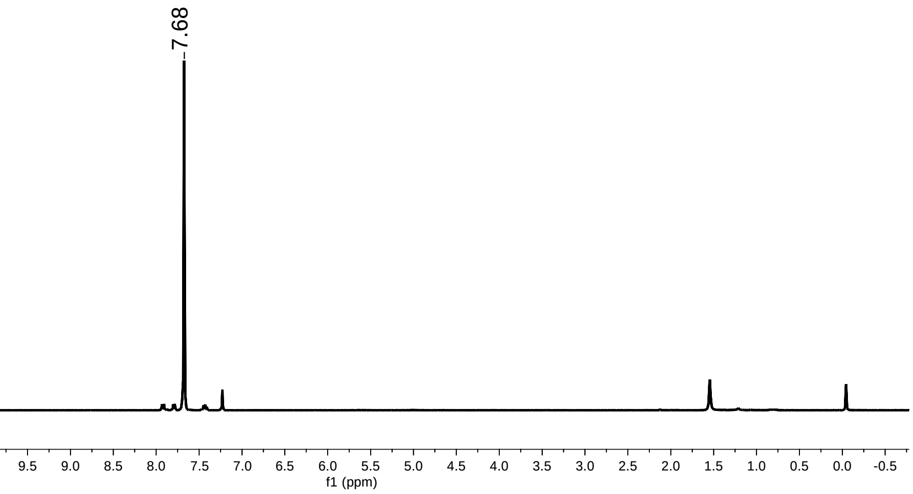


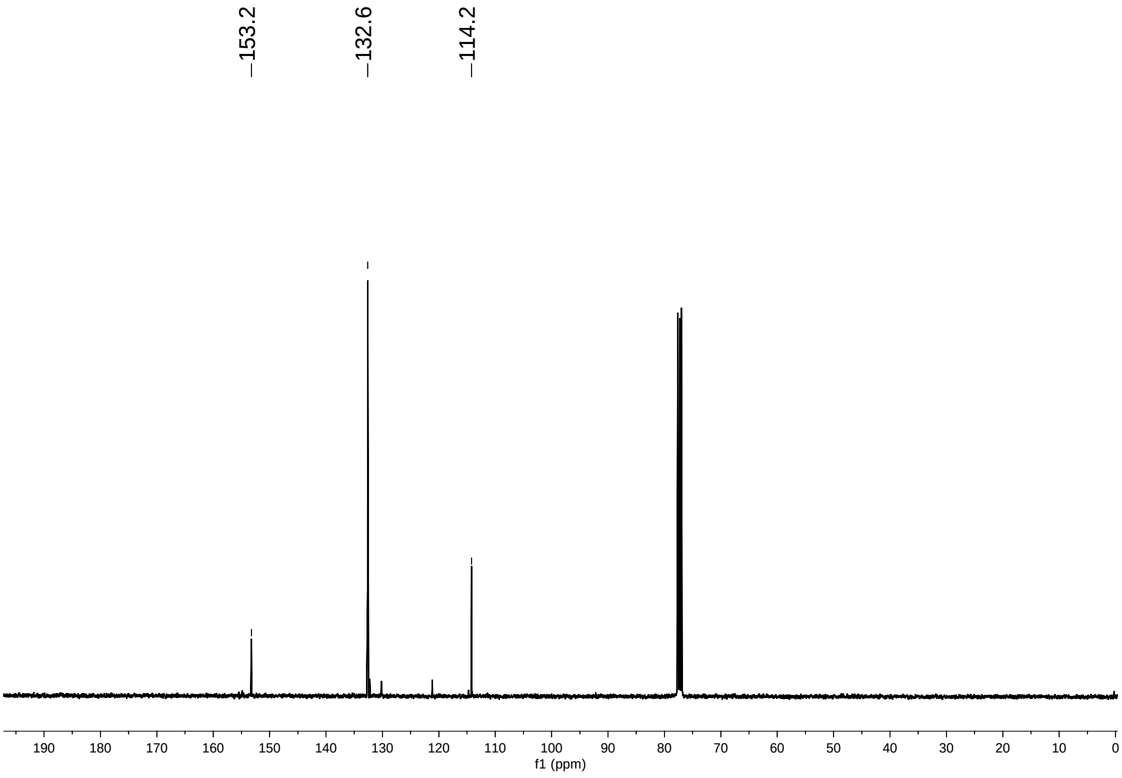

## 2,2-dimethyl-1,2-dihydrobenzo[*a*]furo[2,3-*c*]phenazine (P1)

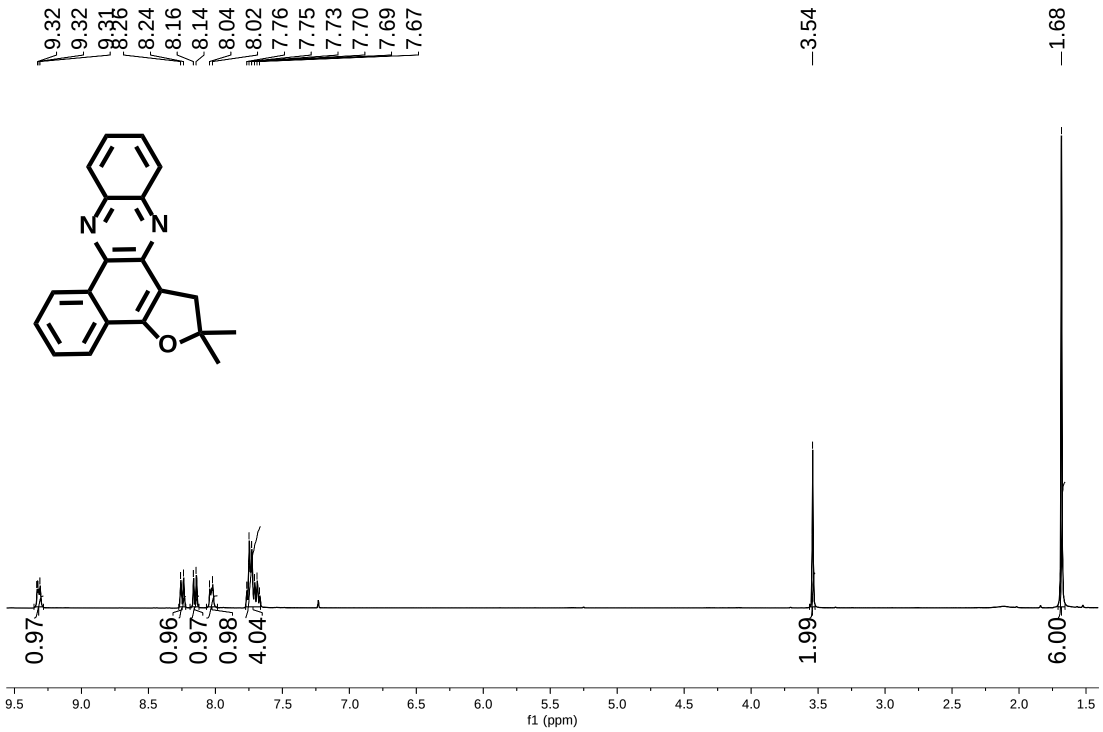


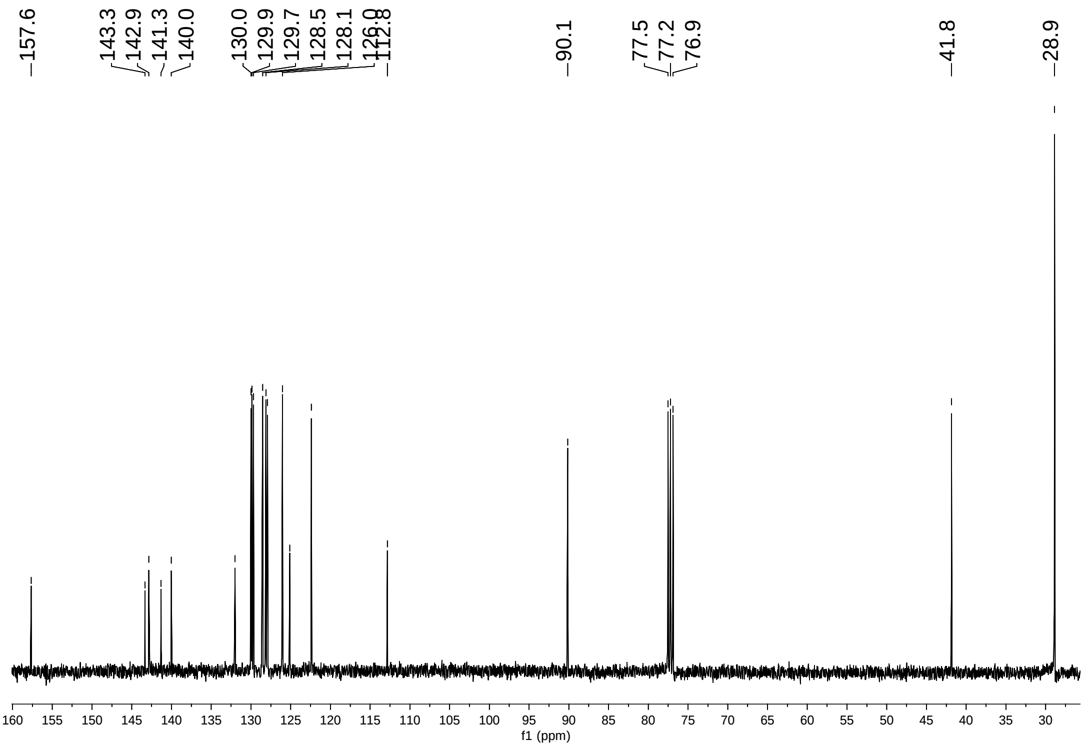

**9,12-dibromo-2,2-dimethyl-1,2-dihydrobenzo[*a*]furo[2,3-*c*]phenazine (P2)**

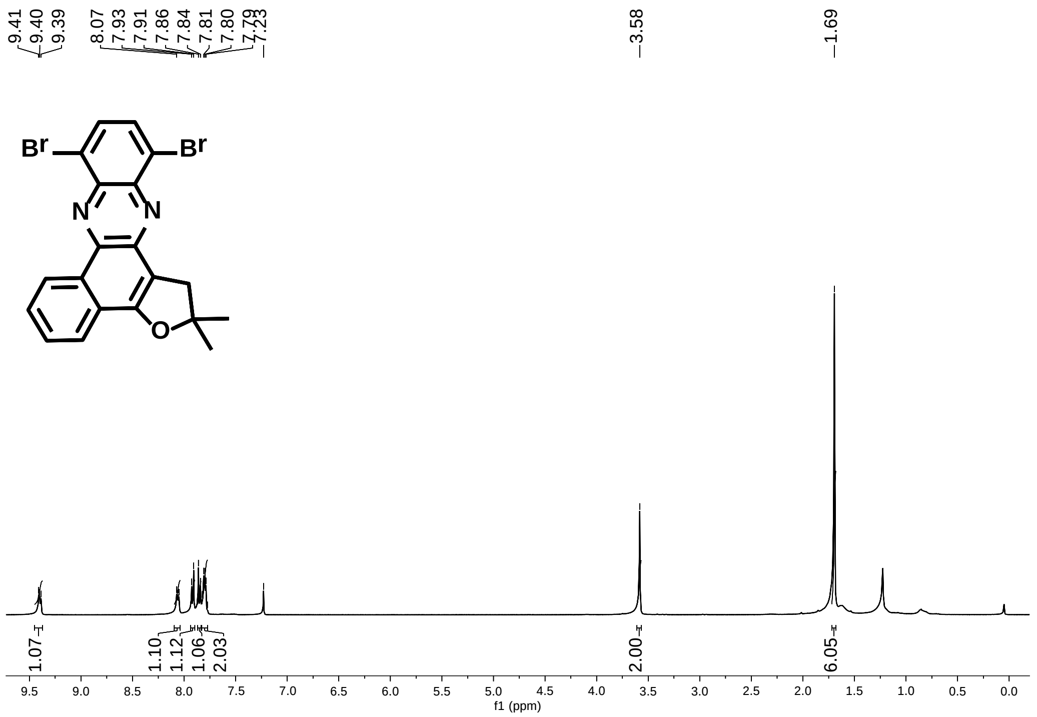


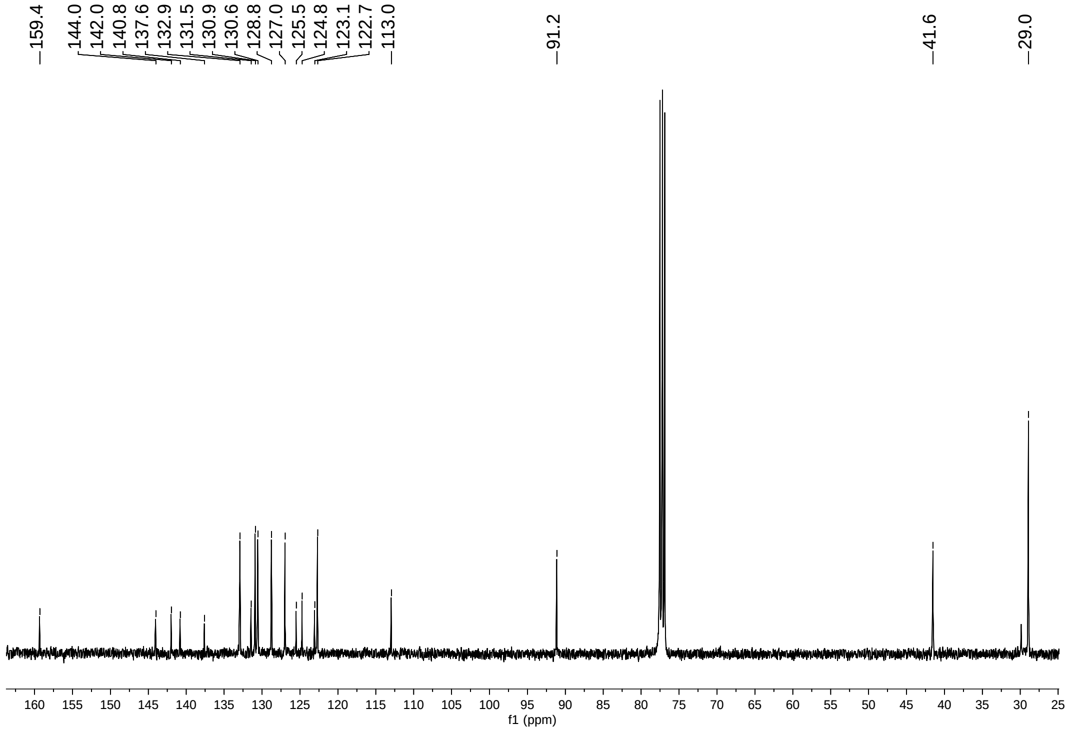

**2,2-dimethyl-9,12-diphenyl-1,2-dihydrobenzo[*a*]furo[2,3-*c*]phenazine (P3)**

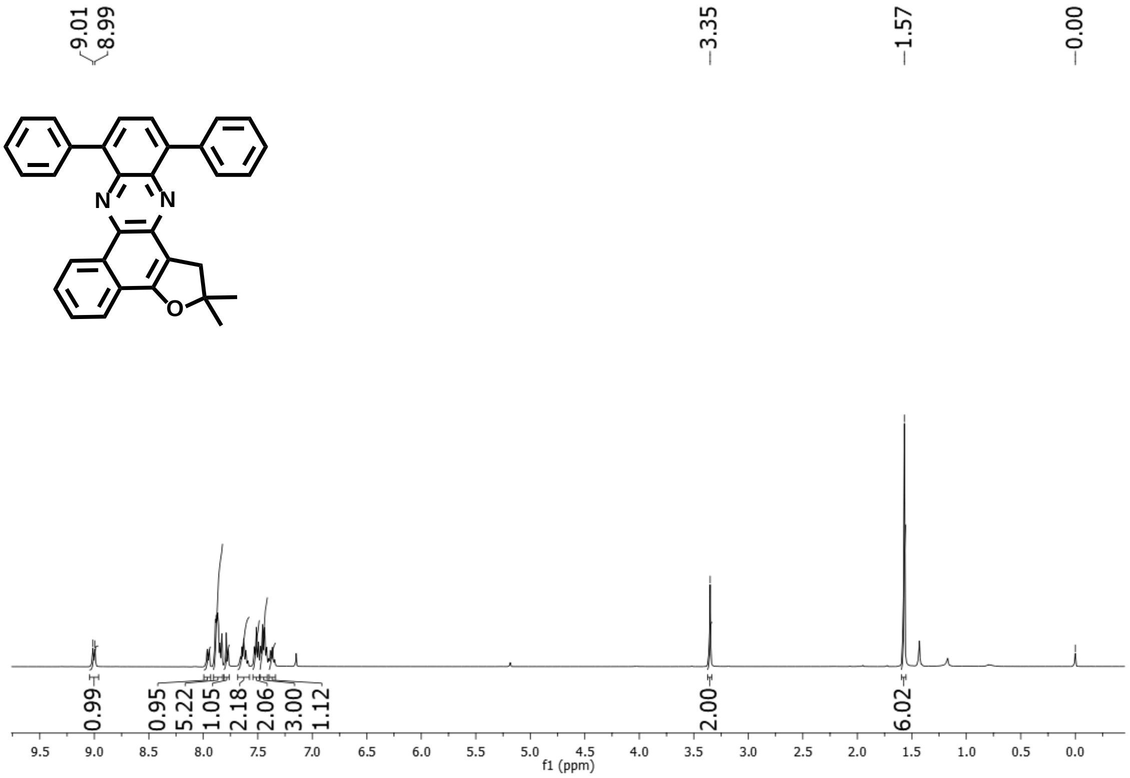


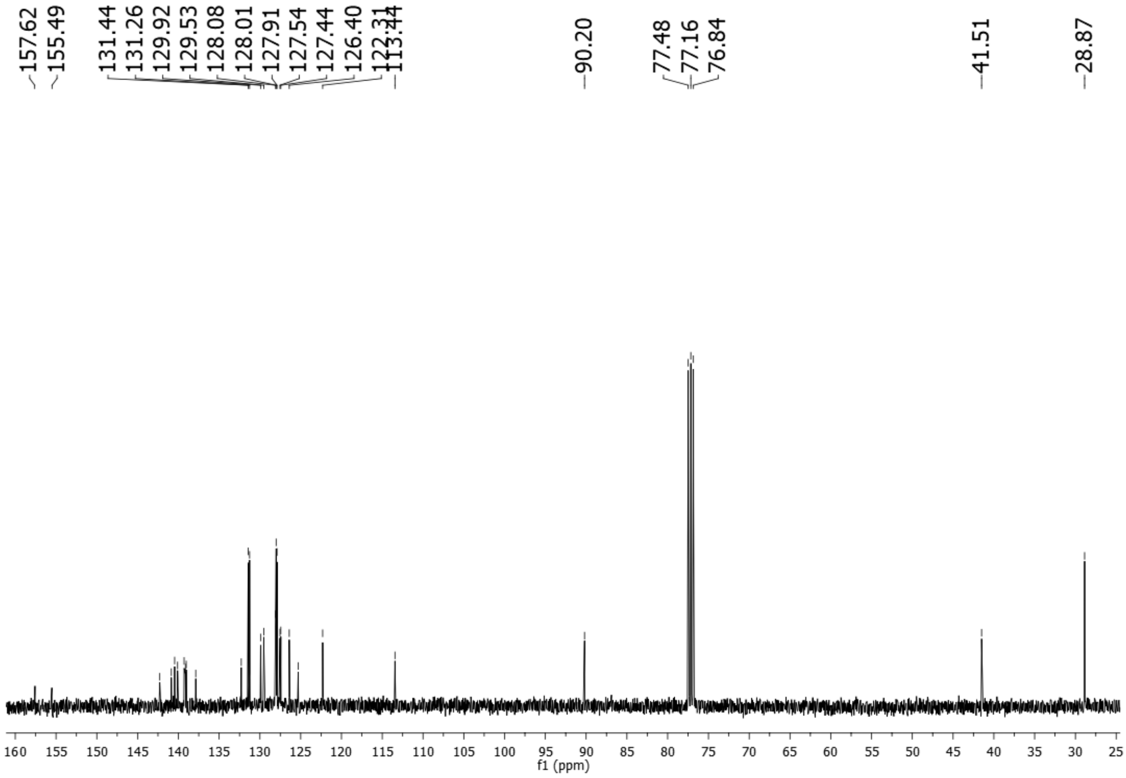

**2,2-dimethyl-9,12-bis((trimethylsilyl)ethynyl)-1,2-dihydrobenzo[*a*]furo[2,3-*c*]phenazine (P4)**

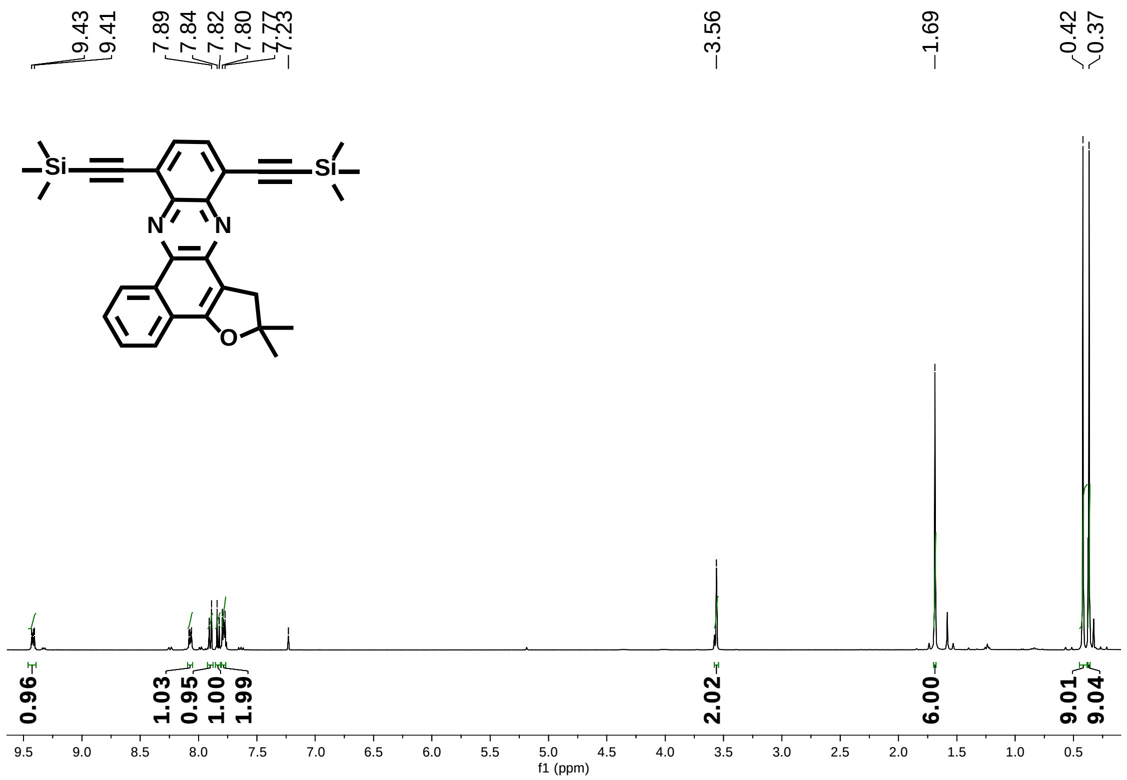


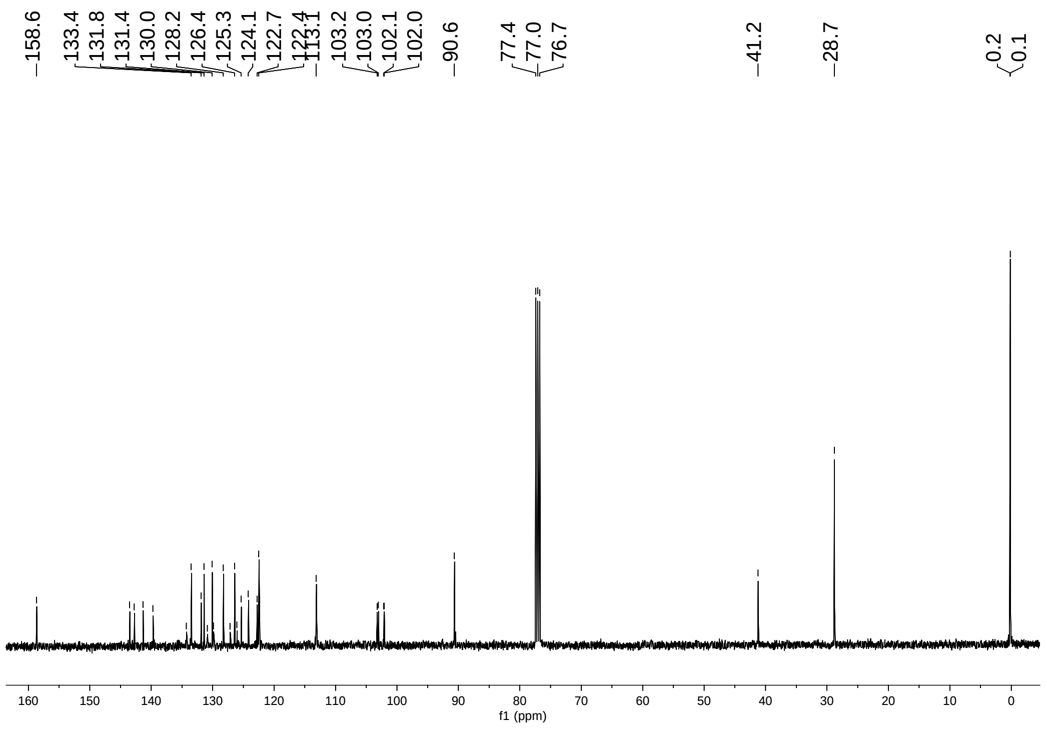

**9,12-diethynyl-2,2-dimethyl-1,2-dihydrobenzo[*a*]furo[2,3-*c*]phenazine (P5)**

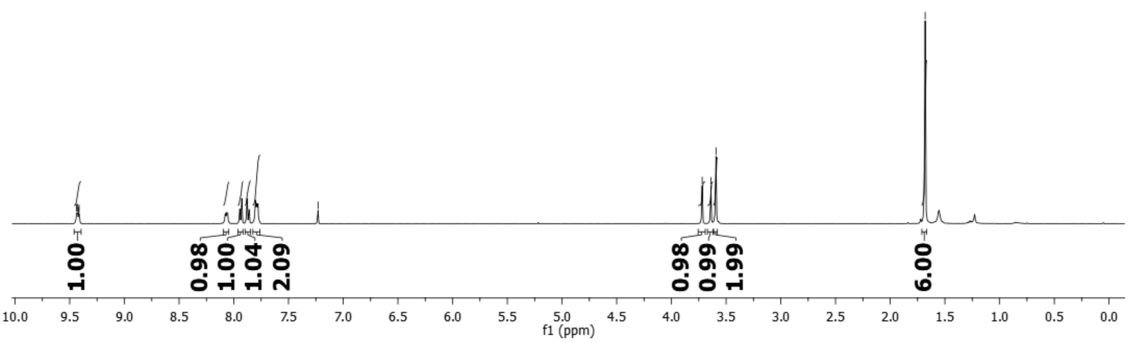


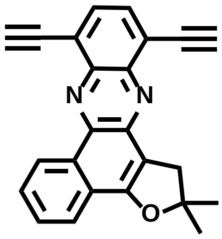


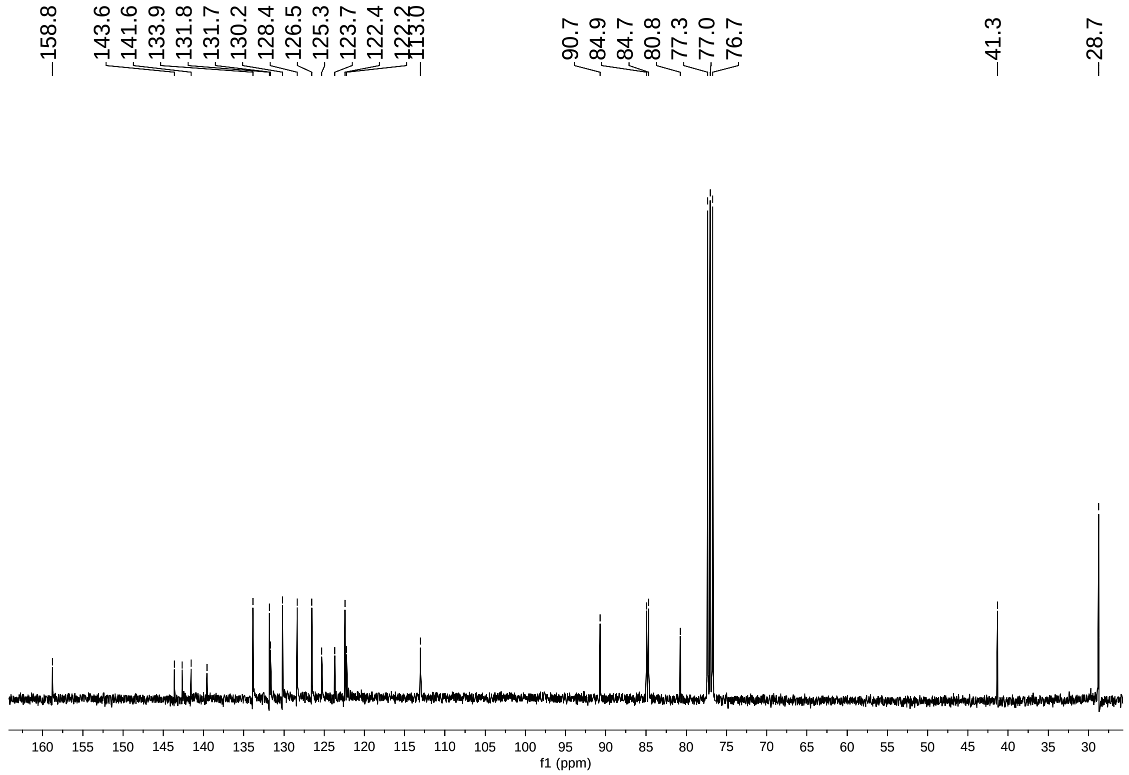

**2,2-dimethyl-9,12-bis(phenylethynyl)-1,2-dihydrobenzo[*a*]furo[2,3-*c*]phenazine (P6)**

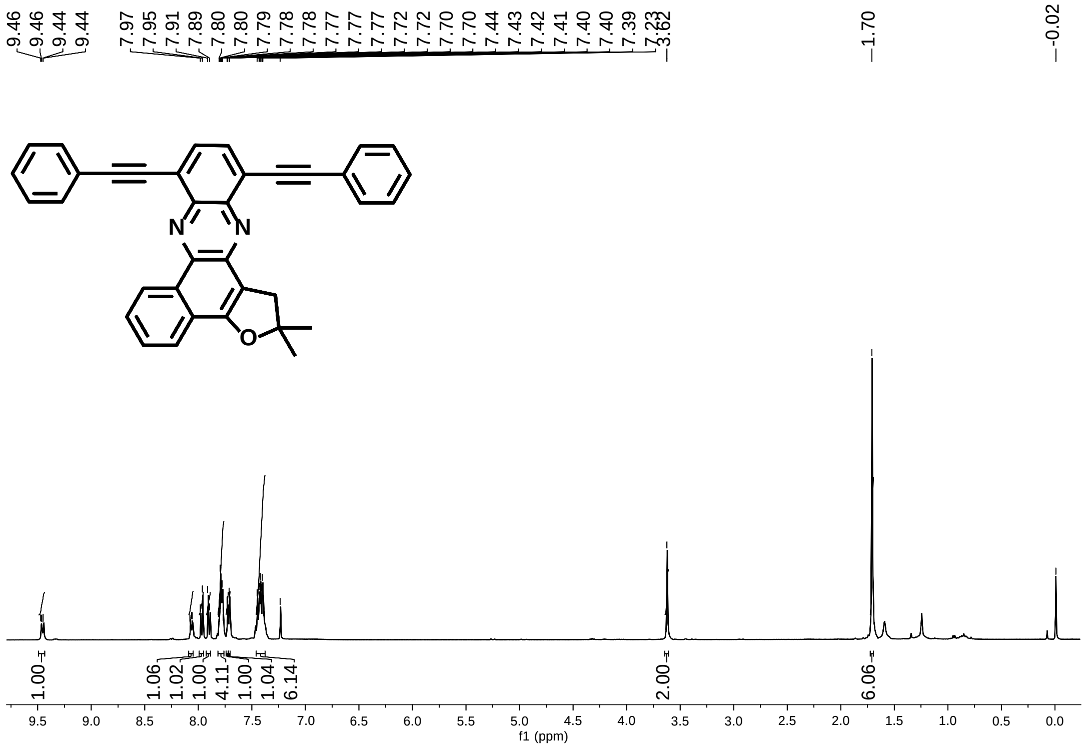


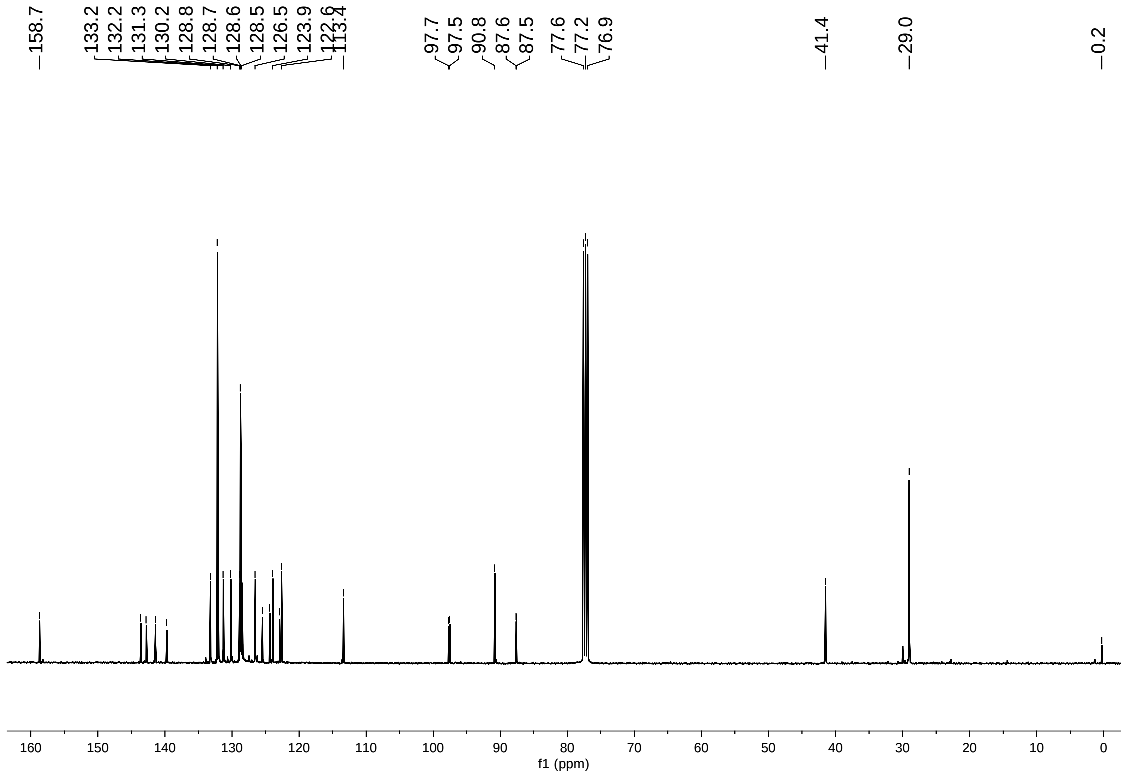

**9,12-bis((4-methoxyphenyl)ethynyl)-2,2-dimethyl-1,2-dihydrobenzo[*a*]furo[2,3-*c*]phenazine (P7)**

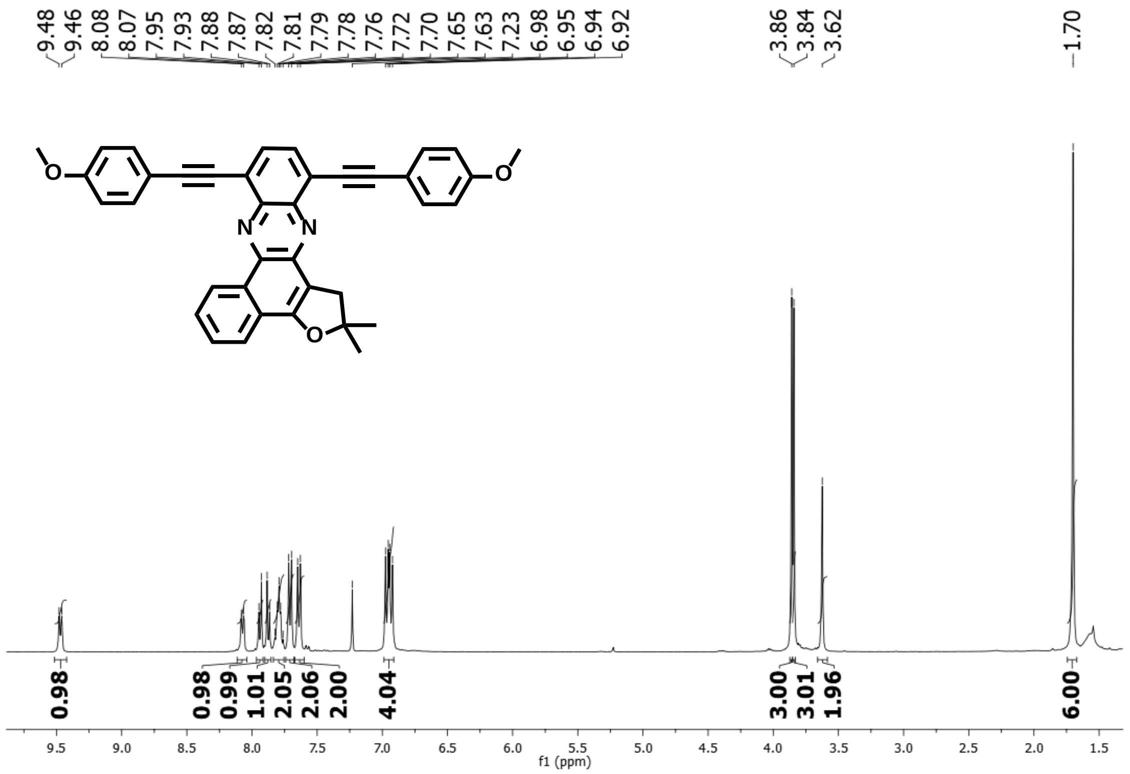

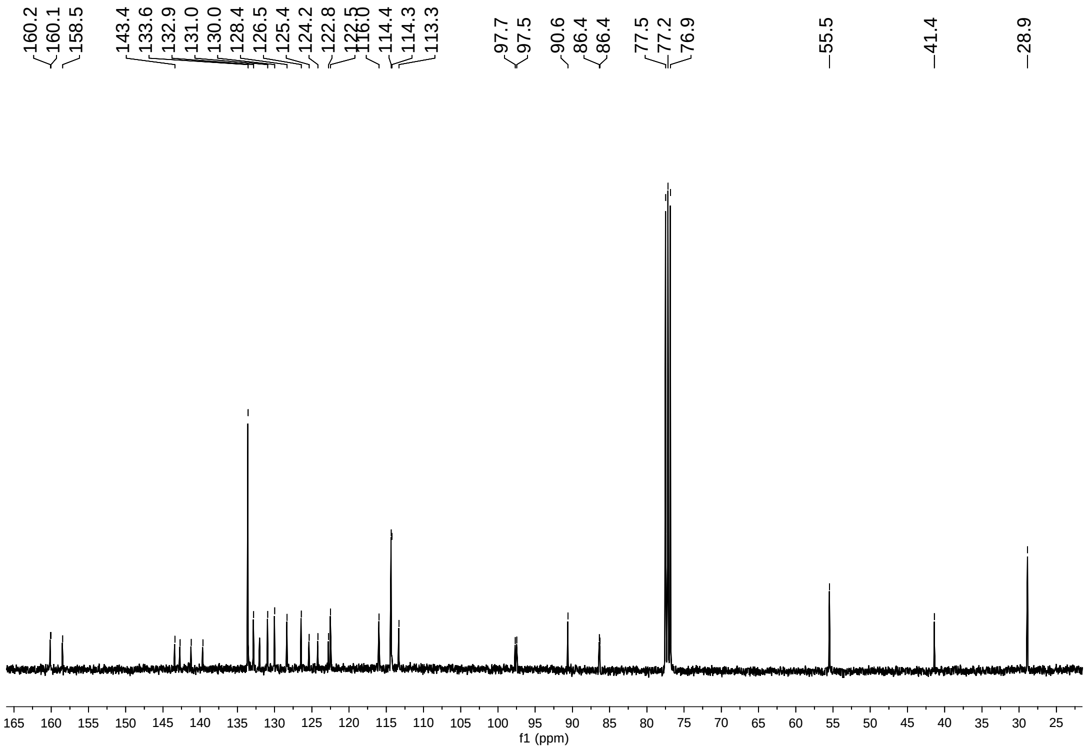

## X-ray Data

**Table S1.** Crystal data and structure refinement of the phenazine **P2**.

| | | |
|---|---|---|
| **Empirical formula** | $C_{20}H_{14}Br_2N_2O$ | |
| **Formula weight** | 458.2 | |
| **Temperature** | 293(2) K | |
| **Wavelength** | 0.71073 Å | |
| **Crystal system** | triclinic | |
| **Space group** | P-1 | |
| **Unit cell dimensions** | a = 7.3251(4) Å | a = 112.91(3)° |
| | b = 10.9814(5) Å | b = 98.21(3)° |
| | c = 11.8736(5) Å | $\gamma$ = 90.81(2)° |
| **Volume** | 868.22(7) $Å^3$ | |
| **Z** | 2 | |
| **Density (calculated)** | 1.75 $Mg/m^3$ | |
| **Absorption coefficient** | 4.67 $mm^{-1}$ | |
| **F(000)** | 452 | |
| **Crystal size** | 0.60 x 0.23 x 0.19 $mm^3$ | |
| **Theta range for data collection** | 2.5 to 27.48° | |
| **Index ranges** | -9≤h1≤8, -13≤k≤14, -15≤l≤15 | |
| **Reflections collected** | 10388 | |
| **Independent reflections** | 3926 [R(int) = 0.06] | |
| **Absorption correction** | none | |
| **Refinement method** | Full-matrix least-squares on $F^2$ | |
| **Data / restraints / parameters** | 3012/ 0 / 226 | |
| **Goodness-of-fit on $F^2$** | 1.020 | |
| **Final R indices [I>2sigma(I)]** | R1 = 0.039, wR2 = 0.056 | |
| **Largest diff. peak and hole** | 0.51 and -0.85 e.$Å^{-3}$ | |

## Supplementary Figures

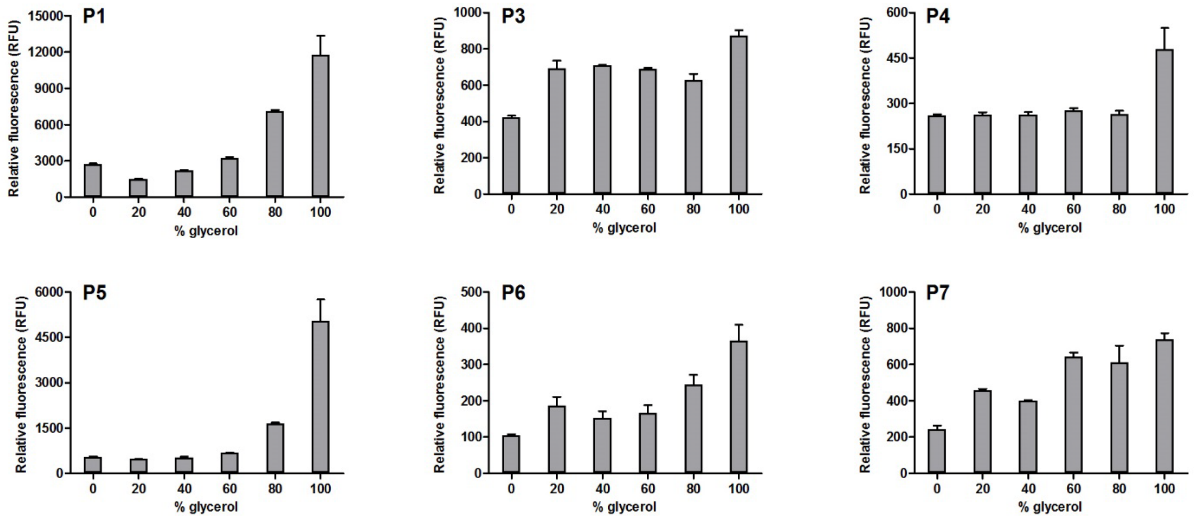


**Figure S1** Fluorescence intensities of phenazine-derived fluorophores **P1** and **P3**-**P7** (50 µM) in water: glycerol mixtures.

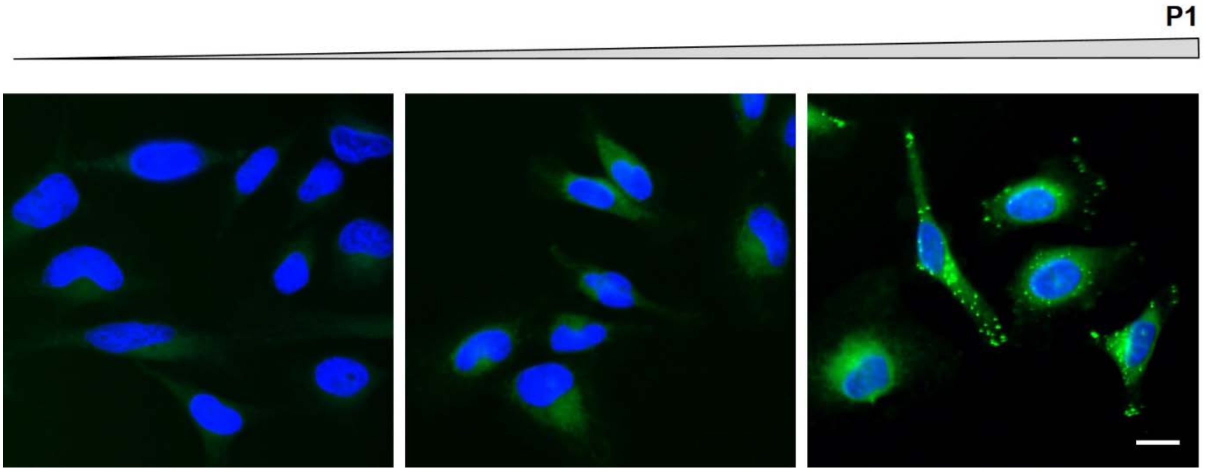


**Figure S2** Fluorescence microscopy images of HeLa cells incubated with compound **P1** (green) at different concentrations: 100 nM (left), 1 µM (center) and 10 µM (right). Cells are counterstained with Hoechst 33342 (blue). Scale bar: 10 µm.

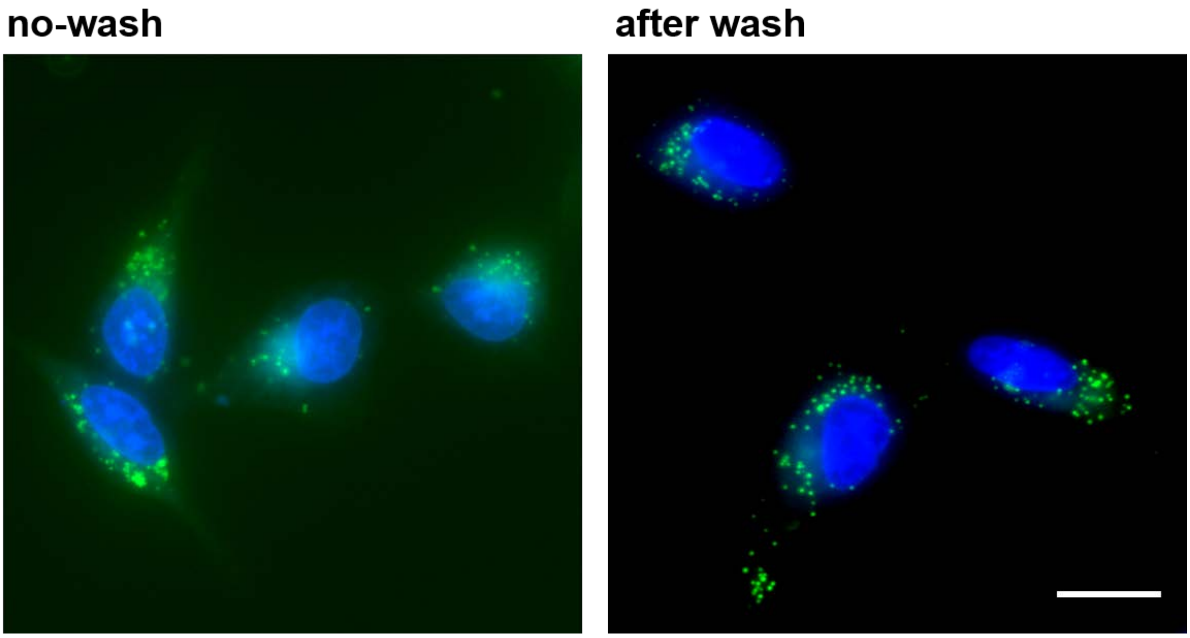


**Figure S3.** High-magnification fluorescence microscopy images of HeLa cells incubated with compound **P1** (10 µM, green) without any washing (left) and after washing. Cells are counterstained with Hoechst 33342 (blue). Scale bar: 10 µm.

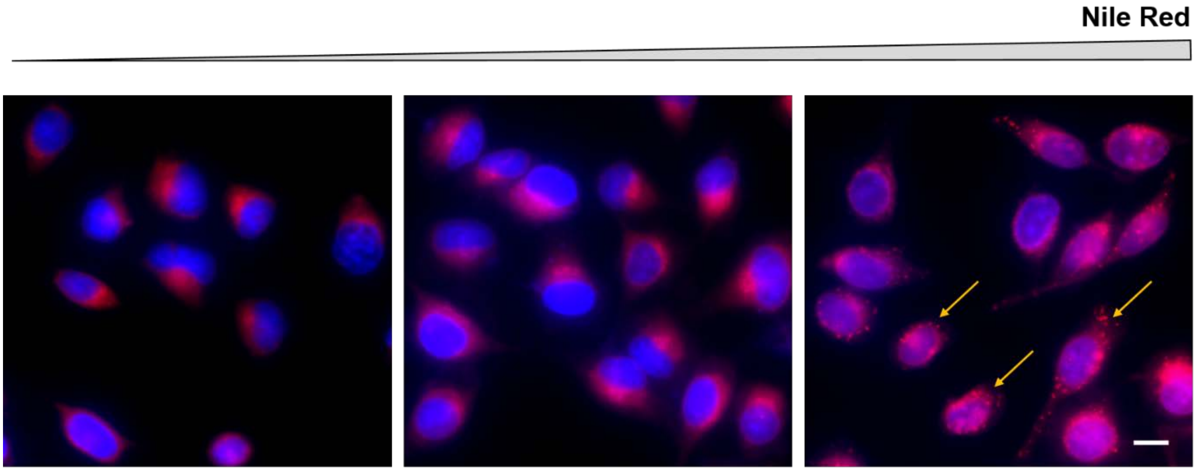


**Figure S4** Fluorescence microscopy images of HeLa cells incubated with Nile red (magenta) at different concentrations: 100 nM (left), 1 µM (center) and 10 µM (right). Cells are counterstained with Hoechst 33342 (blue). Yellow arrows point at LD staining. Scale bar: 10 µm.
